# Circulating cell-free methylated DNA immunoprecipitation sequencing (cfMeDIP-seq) in oncology: from technological advances to clinical applications

**DOI:** 10.1038/s41698-026-01507-w

**Published:** 2026-05-25

**Authors:** Edoardo Francini, Sara Bleve, Giuseppe Nicolo Fanelli, Mara Serena Serafini, Filippo Pederzoli, Alessandra Ferri, Erika Minonne, Jacopo Venturini, Silvia Rodrigues, Hubert Pakula, Cristian Lolli, Massimo Cristofanilli, Pier Vitale Nuzzo

**Affiliations:** 1 Department of Experimental and Clinical Medicine, University of Florence, Florence, Italy; 2 Department of Medical Oncology, IRCCS Istituto Romagnolo per lo Studio dei Tumori (IRST) “Dino Amadori”, Meldola, Italy; 3 Department of Translational Research and New Technologies in Medicine and Surgery, University of Pisa, Pisa, Italy; 4 Department of Pathology and Laboratory Medicine, Weill Cornell Medical College, New York, NY USA; 5 Department of Medicine, Division of Hematology-Oncology, Weill Cornell Medicine, New York, NY USA; 6 Sarah Cannon Research Institute (SCRI), London, United Kingdom

**Keywords:** Biological techniques, Biomarkers, Cancer, Computational biology and bioinformatics, Oncology

## Abstract

Circulating cell-free DNA (cfDNA) methylation profiling enables minimally invasive cancer detection and monitoring. Among available methods, cfMeDIP-seq is a sensitive, scalable, bisulfite-free approach suitable for low-input cfDNA. This review summarizes its principles, comparative technologies, and clinical applications, including early detection, tumor classification, and minimal residual disease monitoring. Despite promising performance, challenges remain in standardization and validation, while emerging multi-omic integrations may further enhance its role in precision oncology.

## Introduction

The discovery that fragments of tumor-derived DNA (ctDNA) circulate in the bloodstream as part of the total circulating cell-free DNA (cfDNA) pool has transformed the field of non-invasive cancer diagnostics^1,2^. cfDNA originates primarily from apoptotic and necrotic cells and mirrors the genomic and epigenomic landscape of the tumor^3^. Over the past decade, cfDNA analysis has become a cornerstone of precision oncology, enabling the detection of somatic mutations, copy-number alterations, and structural variants from a simple blood draw, ushering in the concept of the “liquid biopsy”^4,5^. Compared with tissue biopsy, cfDNA testing offers distinct advantages: it is minimally invasive, captures spatial and temporal tumor heterogeneity, and allows *real-time* disease monitoring. However, mutation-based cfDNA assays alone often fail to detect malignancies with low tumor shedding, limited mutational burden, or extensive subclonal diversity, underscoring the need for complementary biomarkers^6^.

DNA methylation, the covalent addition of a methyl group to the fifth carbon of cytosine (5-mC), represents one of the most stable and biologically informative epigenetic modifications^7,8^. It regulates gene transcription, chromatin organization, and genomic integrity^7,8^. Aberrant methylation, characterized by promoter-specific hypermethylation and genome-wide hypomethylation, is a hallmark of oncogenesis, contributing to tumor-suppressor gene silencing, oncogene activation, and lineage plasticity^9^. Because methylation changes occur early in tumorigenesis and exhibit strong tissue- and cancer-type specificity, they constitute a valuable source of diagnostic, prognostic, and predictive information^10^. Indeed, the consistent and tissue-restricted nature of these epigenetic signatures renders cfDNA methylation profiling particularly attractive for cancer detection, molecular classification, and longitudinal disease monitoring^11,12^.

Traditional cfDNA methylation profiling methods, such as whole-genome bisulfite sequencing (WGBS), reduced-representation bisulfite sequencing (RRBS), and targeted methylation panels, rely on bisulfite conversion to distinguish methylated from unmethylated cytosines^13^. Although bisulfite-based approaches provide single-base resolution, the chemical treatment induces extensive DNA degradation and sequence bias, which represent major limitations when working with the short, low-abundance fragments characteristic of plasma cfDNA^14^. Moreover, these techniques typically require relatively high input quantities and deep sequencing coverage, increasing both cost and technical variability^14^. Such constraints have motivated the development of alternative strategies that preserve cfDNA integrity while providing genome-wide, quantitative methylation profiling in a scalable, clinically adaptable format.

*Circulating cell-free methylated DNA immunoprecipitation sequencing* (cfMeDIP-seq) has emerged as a robust and sensitive enrichment-based alternative^15,16^. This method employs an antibody specific for 5-mC to selectively capture methylated DNA fragments without the need for chemical conversion, thereby preserving fragment integrity and maximizing yield^15,16^. cfMeDIP-seq is compatible with nanogram-level cfDNA inputs and amenable to automation, features that make it particularly well suited for translational and clinical research^15,16^. Proof-of-concept studies have demonstrated that cfMeDIP-seq faithfully reproduces tissue-derived methylation landscapes, discriminates cancer patients from healthy individuals, and accurately infers the tissue of origin (TOO) across multiple tumor types^15,16^.

This review provides a concise overview of cfMeDIP-seq technology, compares it with other cfDNA methylation approaches, highlights its principal clinical applications in cancer detection and monitoring, and discusses ongoing challenges and future directions for its integration into precision oncology.

## Principles and workflow of cfMeDIP-seq

**Fig. 1:**
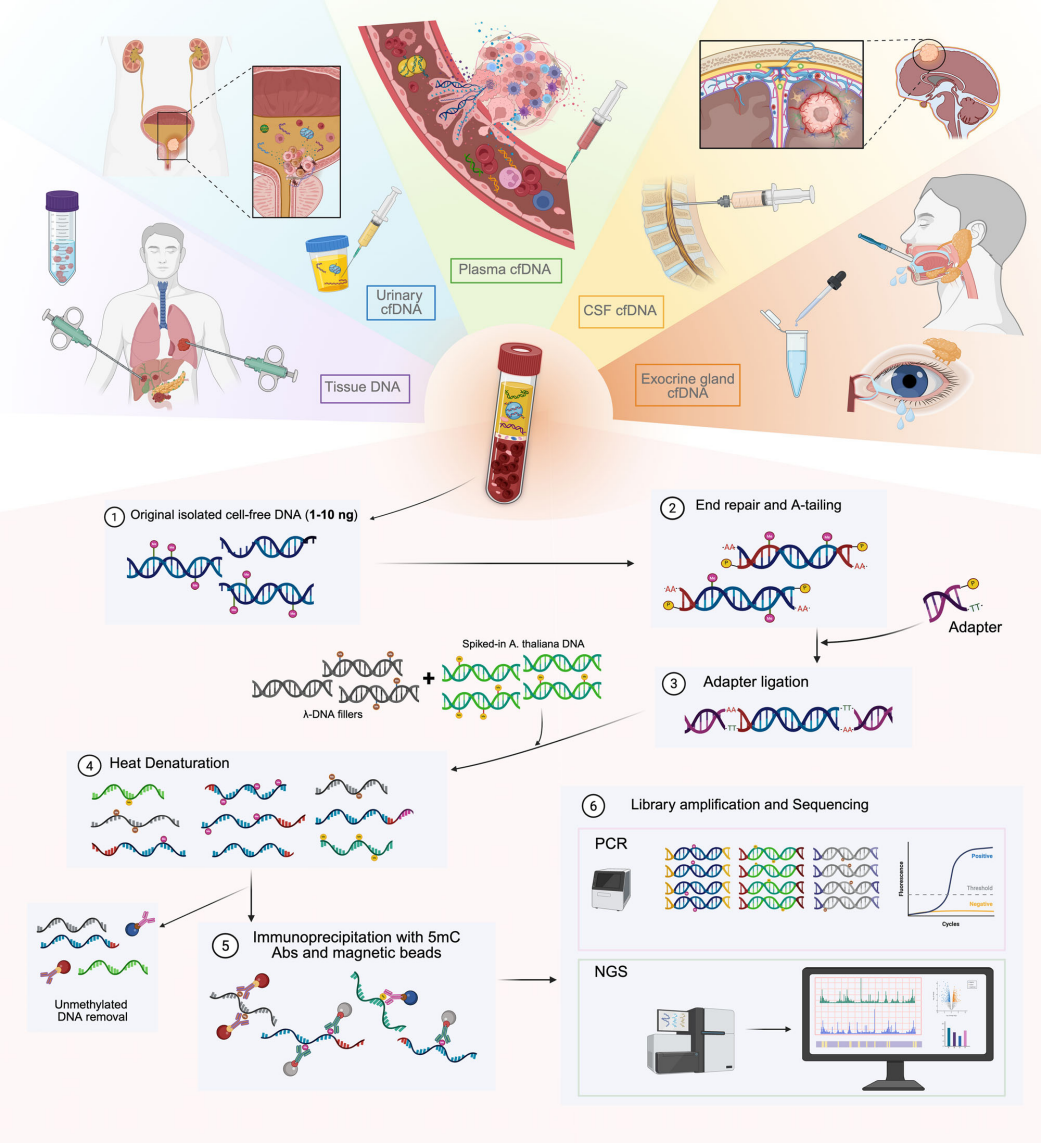
Overview of cfMeDIP-seq and its application to multi-biofluid liquid biopsy. Schematic representation of the biological sources and experimental workflow of circulating cell-free methylated DNA immunoprecipitation sequencing (cfMeDIP-seq). Top panels illustrate the diversity of biological matrices that can be interrogated with cfMeDIP-seq, including plasma, urine, cerebrospinal fluid (CSF), exocrine gland secretions, ocular fluids, and tissue-derived DNA, highlighting the method’s broad applicability across oncologic settings. Bottom panels depict the cfMeDIP-seq workflow. (1) Low-input cfDNA (typically 1–10 ng) is isolated from biofluids. (2) End repair and A-tailing are performed, after which libraries are supplemented with synthetic spike-in controls and λ-DNA filler fragments, followed by (3) ligation of sequencing adapters. (4) Heat denaturation generates single-stranded DNA. (5) Methylated DNA fragments are selectively enriched by immunoprecipitation using 5-methylcytosine-specific antibodies coupled to magnetic beads, while unmethylated DNA is depleted for control. (6) Enriched libraries are PCR-amplified and subjected to next-generation sequencing, enabling genome-wide profiling of methylated cfDNA fragments for downstream bioinformatic analyses, including detection of DMRs, tissue-of-origin inference, and quantitative methylation profiling.

cfMeDIP-seq is an enrichment-based method designed to isolate and sequence methylated cfDNA fragments with high sensitivity and reproducibility^15,16^. By using an antibody specific for 5-mC to capture methylated DNA, this technique preserves fragment integrity and minimizes material loss, an essential advantage when working with the typically short and low-abundance cfDNA. In fact, this approach provides genome-wide coverage while requiring only nanogram-level DNA input, making it particularly attractive for translational and clinical applications (Fig. 1).

The workflow described below reflects commonly adopted practices derived from the original cfMeDIP-seq protocol and subsequent studies^15–21^. Although widely used as a reference framework, these procedures are not yet standardized across laboratories and continue to evolve with ongoing methodological optimization. The cfMeDIP-seq workflow begins with plasma cfDNA extraction using silica- or bead-based methods optimized for nucleosome-sized fragments (approximately 150–250 bp). Accurate evaluation of DNA integrity at this step is essential, as contamination from high-molecular-weight genomic DNA released by leukocyte lysis can dilute the cfDNA signal and compromise data quality. Library construction typically involves end-repair, A-tailing, and adapter ligation using commercially available kits such as NEBNext Ultra II or KAPA HyperPrep. Adapters and indices must be non-methylated to avoid interference with antibody binding, and the use of unique molecular identifiers (UMIs) can reduce PCR bias and help distinguish true duplicates when cfDNA input is particularly low. A portion of the pre-immunoprecipitated DNA is retained as an input control to assess enrichment efficiency and normalize immunoprecipitation yield for downstream analysis.

A distinctive feature of cfMeDIP-seq is its ability to operate effectively with minimal cfDNA input through the inclusion of filler DNA. Filler DNA consists of a mixture of in *vitro-methylated* and *unmethylated lambda (λ)* PCR amplicons, designed to match the size distribution of cfDNA fragments. Acting as a carrier during the immunoprecipitation reaction, filler DNA increases the total DNA available for antibody binding, thereby enhancing the specificity and efficiency of methylated cfDNA recovery. The ratio of methylated to unmethylated filler DNA can be adjusted depending on the experimental context, with commonly reported ratios ranging from 1:1 to 1:5 in the foundational protocol^15^. However, these conditions are protocol-dependent and not universally standardized, and may vary according to workflow design, input material, and analytical objectives. During immunoprecipitation, filler DNA is complemented by synthetic spike-in controls, enabling quantitative calibration. Spike-ins of known methylation content and GC composition are added in precise quantities to correct for immunoprecipitation efficiency, fragment bias, and sequencing depth. These spike-ins enable normalization across experiments and facilitate inter-laboratory reproducibility, although their composition and calibration strategies may differ between studies.

After enrichment, methylated cfDNA is purified, PCR-amplified, and sequenced on next-generation sequencing platforms such as Illumina HiSeq or NextSeq. In several published protocols, sequencing depths of approximately 30–35 million reads per sample have been shown to provide adequate resolution for cancer classification and differential methylation analysis. However, optimal sequencing depth is not standardized and may vary depending on tumor fraction, study design, and required analytical sensitivity. Paired-end sequencing improves mapping accuracy and enables fragment-size determination, which can help distinguish tumor-derived cfDNA from non-tumor-derived fragments.

Rigorous quality control ensures assay specificity and reproducibility. Quantitative PCR of methylated and unmethylated spike-in fragments, such as *Arabidopsis thaliana DNA*, is used to confirm preferential recovery of methylated sequences. The difference in amplification cycles between input and immunoprecipitated fractions should typically fall within three to five cycles, reflecting optimal enrichment. Final libraries are evaluated on a Bioanalyzer to confirm fragment-size distribution and absence of adapter dimers. Sequencing reads are processed through computational pipelines adapted from MeDIP-seq frameworks, although no fully standardized pipeline currently exists, and analytical workflows may vary across studies. After quality control and adapter trimming, reads are aligned using BWA-MEM or Bowtie 2, followed by duplicate removal, fragment counting, and normalization based on spike-in calibration factors. Downstream analyses commonly rely on packages such as MEDIPs or similar frameworks to derive CpG enrichment metrics, genome-wide coverage profiles, and differentially methylated regions (DMRs). Because cfMeDIP-seq captures regional rather than single-nucleotide methylation information, it provides a robust representation of methylation density across CpG-rich domains, promoters, and regulatory elements. Integration with reference methylation atlases enables accurate TOO inference and identification of tumor-specific epigenetic signatures (Fig. 1).

## Alternative cfDNA epigenomic technologies

Several epigenomic technologies have been developed to characterize DNA methylation in circulating nucleic acids, each differing in resolution, input requirements, coverage, and scalability. From a translational perspective, these approaches differ substantially in their balance between analytical resolution and practical feasibility, particularly with respect to cfDNA input requirements, sequencing depth, cost, and scalability in low-input liquid-biopsy settings. Bisulfite sequencing, enzymatic conversion assays, array-based profiling, and affinity-enrichment methods represent the main approaches currently used for cfDNA methylation analysis (Table 1).

**Table 1:** Comparison of cfDNA epigenomic technologies for methylation profiling

Method	Principle	Resolution	cfDNA Input requirement	Coverage	Sequencing burden	DNA integrity	Cost	Scalability	Advantages	Limitations	Applications
WGBS	Bisulfite conversion and high-depth sequencing	Single-base (CpG resolution)	≥10–100 ng	Genome-wide (~28M CpGs)	Very High	Severe DNA damage	Very high	Low	Gold standard for base-resolution mapping; comprehensive coverage	High DNA degradation; high cost and input; complex workflow	Reference-grade methylome mapping; rarely used for cfDNA due to low yield
TAPS	Enzymatic oxidation (TET) and pyridine borane reduction; bisulfite-free	Single-base	10–50 ng	Genome-wide	Very High	Excellent DNA preservation	Very high	Low	Preserves DNA integrity; detects both 5mC and 5hmC	Expensive, multi-step chemistry; low scalability	Experimental use for cfDNA, sensitive detection of 5mC/5hmC
RRBS	Restriction enzyme digestion of CpG-rich regions + bisulfite conversion	Single-base (CpG islands/promoters)	10–100 ng	Partial (~10% genome)	Moderate	Moderate DNA degradation	Moderate	Limited	Cost-effective; enriched for regulatory regions	Enzymatic bias; limited coverage	Targeted methylome studies, promoter methylation profiling
MCTA-seq	PCR amplification of methylated CpG tandem repeats	Locus-specific	≃1–10 ng	Very limited	Low	Good integrity	Moderate	Low	Ultra-sensitive	Narrow genomic scope	Detection of specific methylated biomarkers
Array-Based Profiling	Hybridization of bisulfite-converted DNA to fixed probes	Single-CpG (targeted loci)	200–500 ng (not cfDNA-optimized)	~850,000 CpGs	Low	Poor integrity	Low	High	High reproducibility; cost-effective; standardized	Fixed probe design; low performance with fragmented cfDNA	Biomarker validation; large-scale population studies
EM-seq	Enzymatic oxidation + deamination (bisulfite-free)	Single-base	10–50 ng	Genome-wide	High	Excellent DNA preservation	High	Moderate	Reduced DNA damage; high GC coverage; efficient mapping	Multi-step, costly workflow	Improved integrity for cfDNA methylome profiling
TBS	PCR amplification of selected regions after bisulfite conversion	Single-base (selected loci)	5–20 ng	Limited to the designed targets	Low	Moderate integrity	Moderate	High (targeted)	High sensitivity; quantitative	No genome-wide view	Hypothesis-driven studies; clinical biomarker validation
Methylation-Specific PCR/ddPCR	Allele-specific amplification of methylated/unmethylated DNA	Locus-specific	1–10 ng	Very limited	Very low	Excellent DNA preservation	Low	Very high	Highly sensitive and quantitative	Requires prior target knowledge	Clinical validation and monitoring
MBD-seq	Enrichment of methylated DNA using MBD proteins	Regional (~100 bp)	15–100 ng	Genome-wide (biased to CpG-dense)	Moderate	High DNA integrity	Moderate	Moderate	No chemical conversion; preserves DNA structure	Lower sensitivity in CpG-sparse regions	Profiling methylated regions without damage
cfMeDIP-seq	Immunoprecipitation of methylated cfDNA with anti-5mC antibodies	Regional (~100 bp)	1–10 ng	Genome-wide (non-probe-limited)	Low-moderate	Excellent DNA preservation	Moderate	High	Low input; preserves cfDNA integrity; scalable; robust in low tumor-fraction; quantitative normalization; high sensitivity	No single-CpG resolution; cannot distinguish 5mC/5hmC	Liquid biopsy; DMR detection; tissue-of-origin; tumor profiling; longitudinal monitoring

**Overview of the main epigenomic methods used for cfDNA methylation analysis, compared according to biochemical principle, analytical resolution, cfDNA input requirements, genomic coverage, impact on DNA integrity, cost and scalability, and key advantages, limitations, and applications.**

*WGBS* whole-genome bisulfite sequencing, *TAPS* TET-assisted pyridine borane sequencing, *RRBS* reduced representation bisulfite sequencing, *MCTA-seq* methylated CpG tandem amplification and sequencing, *EM-seq* enzymatic methyl-sequencing, *TBS* targeted bisulfite sequencing, *ddPCR* droplet digital PCR, *MBD-seq* methyl-CpG binding domain sequencing, *cfMeDIP-seq* circulating cell-free methylated DNA immunoprecipitation sequencing, *cfDNA* circulating cell-free DNA, *5mC* 5-methylcytosine, *5hmC* 5-hydroxymethylcytosine, *CpG* cytosine-phosphate-guanine, *DMRs* differentially methylated regions.

WGBS remains the gold standard for base-resolution methylation mapping, enabling interrogation of nearly all CpG sites across the genome^22^. Its main limitations stem from the harsh bisulfite treatment, which causes extensive DNA degradation, a critical issue for fragmented, low-yield cfDNA, and from high input and sequencing depth requirements that increase the cost and complexity of this procedure. Optimizations such as post-bisulfite adapter tagging have improved sensitivity for low-input samples, but their use in cfDNA remains technically demanding^23^. In practice, WGBS requires deep sequencing and relatively high DNA input (typically ≥10–100 ng), amounts that are often difficult to obtain from plasma samples of cancer patients, particularly in low tumor-fraction settings, thereby limiting its applicability in most cfDNA applications.

Ten-eleven translocation (TET) enzyme oxidation of 5mC and 5hmC to 5-carboxylcytosine (5caC)-assisted pyridine borane sequencing (TAPS) offers a bisulfite-free alternative that preserves DNA integrity. TAPS converts modified cytosines while preserving unmodified ones, maintaining four-base genome complexity and improving alignment. Unlike WGBS, TET-based approaches also enable discrimination of 5hmC from 5mC through selective glucosylation. Nevertheless, these processes rely on a slow, multi-step workflow, which substantially increases costs^22,24,25^.

RRBS provides a more economical approach by enriching for CpG-dense regions, such as promoters and CpG islands, via restriction digestion before bisulfite conversion^26^. Although it lowers sequencing costs, RRBS sacrifices genome-wide coverage, typically interrogating only 10–20% of the genome^26–28^. PCR-based methods like methylated CpG tandem amplification and sequencing achieve picogram-level sensitivity but remain limited in genomic scope^29^. Coverage is largely restricted to CpG-dense regions (≥3 CpG/100 bp), omitting distal regulatory elements and CpG-sparse regions relevant for applications such as TOO inference and biomarker discovery. Notably, DNA input requirements remain relatively high (10–100 ng).

Array-based technologies such as the Illumina Infinium MethylationEPIC v2.0 enable targeted, high-throughput profiling of more than 935,000 CpG sites, profiling key regulatory regions (including ENCODE and FANTOM5 enhancers), as well as genomic loci designed for CNV detection and over 450 cancer driver mutations^30–32^. They are cost-effective, reproducible, and potentially useful for biomarker validation and population-scale studies^30^. A major issue is their high DNA input requirements (up to 1 µg). Empirical assessments have shown that reducing the input to 125–250 ng leads to a significant increase in failed probes and compromised data quality^32^.

Enzymatic methyl-seq (EM-seq) replaces bisulfite conversion with enzymatic oxidation and deamination, preserving DNA integrity and supporting library inserts up to ~350 bp^33^. However, recent evidence highlighted important practical limitations^34^. The multiple magnetic bead clean-up steps required between enzymatic reactions result in low final DNA recovery rates (34-47%), compared with standard bisulfite-based protocols (61–81%), thereby impairing their utility in low-yield samples. In addition, the standard EM-seq protocol does not distinguish between 5mC and 5hmC.

On the other hand, targeted bisulfite sequencing (TBS) and complementary PCR-based assays such as methylation-specific PCR and droplet digital PCR enable focused, high-sensitivity interrogation of selected loci, achieving limits of detection up to 0.001%^35^. However, targeted approaches have limited multiplexing capacity, restricting analysis to a small number of loci per assay and making them suitable mainly for hypothesis validation. Their reliance on a priori knowledge precludes the genome-wide breadth required for de novo biomarker discovery and complex TOO inference.

Finally, unlike chemical- or enzymatic-conversion-based methods, affinity-enrichment approaches are novel technologies that use antibodies or binding proteins to selectively capture methylated DNA fragments without compromising cfDNA integrity. These strategies lack single-CpG resolution, but are designed to isolate entire methylated DNA fragments, generating a regional signal across genomic loci^36,37^.

MeDIP-seq and methyl-CpG binding domain-sequencing (MBD-seq) are two affinity-enrichment approaches for DNA methylation profiling, which differ in terms of biochemical basis and genomic coverage^37–39^. MBD-seq uses methyl-CpG binding domain (MBD) proteins (e.g., MBD2) to bind methylated double-stranded DNA (dsDNA). Its binding affinity depends on the presence of multiple methylated cytosines in close proximity, resulting in a strong bias toward CpG-dense regions such as CpG islands and reduced efficiency in capturing sparsely methylated regions. In addition, it requires relatively high DNA input (15–100 ng)^36,37^. Conversely, MeDIP-seq leverages a monoclonal antibody specific for 5mC to selectively capture methylated DNA fragments, enabling coverage of regions with sparsely methylated CpG sites^15,20^. This feature makes it particularly suitable for applications such as TOO identification^37–39^. Compared with base-resolution approaches, cfMeDIP-seq enables accurate methylation profiling from low DNA input (1–10 ng) while preserving fragment integrity, making it particularly well-suited for liquid biopsy longitudinal monitoring and early detection applications^15,16^. Importantly, this method has shown high concordance with both standard MeDIP-seq and bisulfite-based approaches such as RRBS, demonstrating robust CpG enrichment and high reproducibility even at clinically relevant cfDNA input levels^15,16^.

Additionally, comparative analysis of regional methylation profiles across the genome enables the identification of DMRs. DMRs represent epigenomic patterns that serve as robust biomarkers for distinguishing malignant from normal tissues and for accurately inferring TOO in clinical settings, as demonstrated in multiple studies^15,16,19,20,40,41^.

Overall, despite lacking single-CpG resolution and failing to distinguish 5mC from 5hmC, cfMeDIP-seq offers a favorable balance among genome-wide coverage, low input requirements, and scalability, while maintaining sufficient resolution to capture biologically meaningful methylation patterns in liquid-biopsy samples.

## Applications of cfMeDIP-seq in cancer

The capacity of cfMeDIP-seq to generate high-fidelity methylation profiles from minimal cfDNA input is enabling its rapid adoption in oncologic research, supporting applications that span disease classification, early detection, residual disease monitoring, and treatment response assessment (Table 2). Importantly, the strength of evidence supporting these applications is not uniform. While some studies include relatively large cohorts, independent validation sets, or multicenter designs, a substantial portion of the literature remains based on retrospective, proof-of-concept investigations with limited sample sizes, selected patient populations, and variable stage distributions. Accordingly, reported performance metrics should be interpreted in the context of study design and validation maturity.

**Table 2:** Clinical applications of cfMeDIP-seq across tumor types

Study/Year	Tumor types(s)	Setting	Number of samples	Performance	Validation	Study design	Analytical approach
**Cancer detection/early detection**							
Shen et al., Nature 2018	Pancreatic ductal adenocarcinoma (PDAC), lung cancer (LC), breast cancer (BC), colorectal cancer (CRC), renal cell carcinoma (RCC), urothelial bladder cancer (UBC), and acute myeloid leukemia (AML), plus healthy controls	Tumor classification/tissue-of-origin inference	388 total plasma samples (189 discovery cohort, 199 validation cohort) across multiple tumor types and controls	AUC = 0.98 (AML vs others), 0.92 (PDAC vs others), 0.97 (lung vs others), 0.97 (cancer vs normal)	Internal validation (independent validation cohort)	Retrospective, case-control	cfMeDIP-seq
Nuzzo et al., Nat Med 2020	Renal cell carcinoma (RCC); urothelial bladder cancer (UBC); controls	Cancer Detection/Tumor classification	148 samples (99 RCC, 21 UBC, 28 healthy controls); plasma and urine cfDNA	Plasma: AUROC 0.99 (RCC vs controls); AUROC 0.979 (RCC vs UBC). Urine: AUROC 0.858	Internal (repeated train-test splits, 100 iterations)	Retrospective, case-control	cfMeDIP-seq
Nassiri et al., Nat Med 2020	Brain tumors (glioma, meningioma, hemangiopericytoma, brain metastases)	Tumor classification/tissue-of-origin inference	220 intracranial tumor patients (70 IDH-mut glioma, 52 IDH-wt glioma, 60 meningioma, 9 hemangiopericytoma, 14 glioneuronal tumors, 15 brain metastases) + healthy controls; total cfMeDIP cohort: 447 plasma samples	AUC 0.99 (glioma vs others, 95% CI, 0.96–1.00); subclass AUCs: meningioma 0.89, hemangiopericytoma 0.95, low-grade glial-neuronal 0.93, IDH-mutant glioma 0.82, IDH-wildtype glioma 0.71	Internal cross-validation	Retrospective	cfMeDIP-seq with machine-learning classification based on genome-wide methylation profiles
Lasseter et al., Genet Med 2020	Metastatic renal cell carcinoma (mRCC)	Cancer detection/method comparison (cfMeDIP-seq vs cfDNA variants)	40 mRCC patients (variant analysis cohort); 34 mRCC patients with cfMeDIP-seq; independent training cohort: 38 RCC patients + 34 cancer-free controls	cfMeDIP-seq: sensitivity 100% (34/34), specificity 88% (controls), AUC 0.98; cfDNA variant detection: 21% (7/34)	Internal (training/test split; no external validation)	Retrospective	cfMeDIP-seq classifier based on genome-wide DMRs (vs targeted cfDNA sequencing)
Qi et al., Cancer Science 2021	Lung cancer (LC)	Cancer Detection	97 samples (67 lung cancer, 30 healthy controls)	AUROC 0.963; sensitivity 91.0%, specificity 93.3% (lung cancer vs healthy controls)	Not reported	Retrospective, case-control	cfMeDIP-seq
Lu et al., Clin Epigenetics 2022	Ovarian cancer	Cancer Detection	180 plasma samples (74 ovarian cancer, 86 healthy controls, 20 benign ovarian disease)	AUC 0.97 (ovarian vs controls); sensitivity 93%, specificity 91%	Internal (train/test split)	Retrospective	cfMeDIP-seq with a machine-learning classifier based on genome-wide DMRs
Zhang et al., World J Surg Oncol 2022	Colorectal cancer (CRC)	Cancer detection (exploratory biomarker discovery)	7 plasma samples (4 CRC, 3 healthy controls); additional validation in public tissue datasets (*n* = 488)	AUC 0.928 (training), 0.915 (validation) based on TCGA/GEO tissue methylation data (12-probe signature)	External validation (tissue datasets only)	Retrospective, exploratory study	MeDIP-seq (cfDNA discovery + tissue-based model validation)
Wang, J., et al., Epi & Chromatin 2023	Breast cancer	Cancer Detection	49 plasma samples (breast cancer + healthy controls; discovery and validation cohorts)	AUROC 0.909 (validation cohort); sensitivity 63.6%, specificity 100% using DMR-dependent fragmentation score	Internal validation cohort	Retrospective study	cfMeDIP-seq (integrated methylation and fragmentation analysis)
Qi et al., Cancer Science 2024	Gastric cancer (GC)	Cancer Detection	250 samples (150 GC, 100 healthy controls)	AUROC: 0.9877 (discovery) and 0.9802 (validation); sensitivity 93.9–88.4%, specificity 95.2–94.2%	Internal validation	Retrospective, case-control	cfMeDIP-seq
Terp et al., Cancers 2025	Ovarian cancer (OC); benign ovarian conditions; healthy controls	Tumor classification/cancer detection	116 samples (40 OC, 38 benign, 38 healthy controls)	Distinct cfDNA hypermethylation signature across 15 genes; consistent differential methylation between OC and controls	No independent validation	Retrospective, exploratory study	cfMeDIP-seq
Zeng et al., Nature Cancer 2026	Brain cancer, lung cancer (LC), prostate cancer (PC), acute myeloid leukemia (AML), pancreatic ductal adenocarcinoma (PDAC), uveal melanoma (UM), head and neck squamous cell carcinoma (HNSCC), breast cancer (BC), colorectal cancer (CRC), urothelial bladder cancer (UBC), renal cell carcinoma (RCC); healthy controls; Li-Fraumeni syndrome (LFS)	Cancer detection/tumor classification (pan-cancer)	1,074 plasma cfMeDIP-seq samples (918 individuals): brain (*n* = 161), lung (*n* = 141), prostate (*n* = 133), AML (*n* = 78), PDAC (*n* = 71), UM (*n* = 46), HNSCC (*n* = 35), BC (*n* = 25), CRC (*n* = 23), UBC (*n* = 20), RCC (*n* = 20), healthy (*n* = 153), LFS (*n* = 168); validation cohort: 220 independent samples; total = 1294	Integrated methylation and fragmentomic features improved cancer detection and classification (mAUC up to ~0.99)	Independent validation cohort (*n* = 220)	Retrospective multi-cohort integration	cfMeDIP-seq + fragmentomics
**Tumor classification/tissue of origin**							
Baca et al., Nat Med 2023	Advanced solid tumors (breast, lung, prostate, esophageal, Merkel cell carcinoma, melanoma, glioma)	Tumor classification/molecular subtyping	1,268 plasma cfDNA samples from patients with advanced solid tumors across seven cancer types and controls (multi-cohort dataset)	NEPC vs PRAD AUC 0.97; NE vs non-NE AUC 0.94; BC ER+ vs ER− AUC 0.90; SCLC vs NSCLC AUC 0.90; Merkel cell vs non-NE cancers AUC 0.90	Internal validation	Retrospective, multi-cohort study	cfMeDIP-seq
Berchuck et al., Clin Cancer Res. 2022	Neuroendocrine prostate cancer (NEPC) vs CRPC adenocarcinoma (PRAD)	Cancer detection/tumor subtype classification	101 total cfDNA samples (21 NEPC, 80 CR-PRAD); test cohort: 48 (9 NEPC, 39 PRAD); validation cohort: 53 (12 NEPC, 41 PRAD)	AUROC = 0.96 (test cohort); AUROC = 1.00 (independent validation cohort)	External validation (independent cohort)	Retrospective, case-control	cfMeDIP-seq (tissue-informed DMR-based classifier)
Chen et al., Nat Commun 2022	Prostate cancer (localized and metastatic) Tumor classification/cancer detection	Tumor classification	235 plasma samples (prostate cancer patients and healthy controls)	AUC = 0.989 (localized vs metastatic PC); Sens ≈ 0.99; Spec ≈ 0.99	Internal (cross-validation)	Retrospective, case-control	cfMeDIP-seq with machine-learning classifier (genome-wide methylation features)
Zuccato et al., Nat Med 2023	Brain metastases	Prognostic stratification/tumor classification	123 plasma samples (brain metastases and other cancers/controls)	AUROC 0.80 (95% CI, 0.68–0.93, brain metastases vs others)	Internal validation	Retrospective study	cfMeDIP-seq
Haq et al., iScience 2022	Small-cell lung cancer (SCLC)	Tumor classification/prognostic stratification	94 plasma samples (74 SCLC, 20 non-cancer controls)	Two methylation-defined clusters associated with OS (HR = 2.02, *p* = 0.014); median OS: 21 vs 13 months	Internal (no independent validation cohort)	Retrospective, observational	cfMeDIP-seq with leukocyte methylation subtraction (PRIME algorithm) and unsupervised clustering
Grisolia et al., Clin Epigenetics 2025	Breast cancer (BRCA1/2mut)	Tumor classification/cancer detection	44 plasma samples (23 BRCA1/2-mut BC, 19 BRCA1/2-wt controls, 2 healthy controls)	AUC = 0.95 (BRCA1/2-mut BC vs controls); Sens = 88%; Spec = 94.7%	Internal (no independent external cohort)	Retrospective, case-control	cfMeDIP-seq with BRCA-associated methylation signature
**Minimal residual disease/treatment monitoring**							
Peter et al., Epigenomics 2020	Metastatic castration-resistant prostate cancer (mCRPC)	Treatment monitoring/response prediction	45 plasma cfDNA samples (longitudinal) from mCRPC patients	Longitudinal cfDNA methylation changes associated with time to progression; sustained methylation alterations correlated with better response; NE-CRPC signatures linked to worse outcomes	No independent validation	Retrospective, longitudinal	cfMeDIP-seq
Burgener et al., Clin Cancer Res 2021	HPV-negative head and neck squamous cell carcinoma (HNSCC)	Cancer detection/treatment monitoring/prognostic stratification	77 plasma samples (30 HNSCC patients, 20 healthy controls, matched leukocyte controls)	AUC = 0.94 (HNSCC vs healthy); Sens = 0.90; Spec = 0.89; ctDNA detection associated with worse OS	Internal (no independent external cohort)	Retrospective, case-control	cfMeDIP-seq combined with mutation-based ctDNA analysis (multimodal plasma profiling)
Janke et al., Clin Epigenetics 2022	ALK-positive non-small-cell lung cancer (NSCLC)	Treatment monitoring/prognostic stratification	79 plasma samples (66 from 21 ALK + NSCLC patients, 13 healthy controls; longitudinal sampling)	5mC score correlated with tumor burden (*ρ* > 0.6); higher scores associated with worse OS (*p* = 0.025); rising methylation preceded radiologic progression in 7/8 cases	Internal (longitudinal analysis, no external validation cohort)	Retrospective, longitudinal	cfMeDIP-seq with tumor-informed DMR selection and background signal correction using WGBS reference datasets
Ravera et al., J Liquid Biopsy 2024	Breast cancer (early, triple-positive)	Treatment monitoring/response prediction	7 patients; longitudinal plasma cfDNA samples (baseline, end of NACT, post-surgery)	Distinct methylation dynamics between pCR and RD patients; significant DMR changes observed only in responders; PCA separated response groups; potential for MRD detection	No independent validation	Retrospective, pilot study	cfMeDIP-seq
Liu et al., Ann. Oncol. 2024	Head and neck cancer (HNSCC, HPV+ and HPV−)	Molecular residual disease (MRD) detection/treatment monitoring	325 patients, 1155 plasma samples (longitudinal; training and independent validation cohorts)	MRD positivity strongly associated with worse RFS (HR = 35.7, *p* < 0.0001); sensitivity 91%, specificity 88%; lead time to recurrence up to 14.9 months	Independent blinded validation cohort	Retrospective, longitudinal (training + validation design)	cfMeDIP-seq with genome-wide tissue-agnostic methylation classifier for MRD detection
Zhao et al., Cancer Discovery 2024	Multiple solid tumors (TNBC, HGSOC, HNSCC, melanoma, others)	Treatment monitoring/response prediction (ICB)	204 plasma samples from 87 patients (INSPIRE trial; longitudinal sampling)	Early changes in cfMeDIP-seq methylation and fragmentation predict response and survival (correlation with ctDNA and OS/PFS)	Internal validation	Prospective clinical trial (Phase II, INSPIRE)	cfMeDIP-seq (tumor-naïve methylation + fragmentomics integration)
Stutheit-Zhao et al., npj Precision Oncology 2026	Head and neck squamous cell carcinoma (HNSCC), triple-negative breast cancer (TNBC), high-grade serous ovarian cancer (HGSOC), melanoma (MEL), mixed solid tumors (MSTs)	Treatment monitoring/response prediction (anti-PD-1, pembrolizumab)	106 patients enrolled; 69 evaluable; 241 plasma cfDNA samples (longitudinal). Tumor distribution (enrolled): TNBC (*n* = 22), HGSOC (*n* = 21), HNSCC (*n* = 19), MEL (*n* = 12), MST (*n* = 13)	Early changes in cfDNA methylation score predicted response, PFS, and OS	Blinded validation	Prospective, phase II	cfMeDIP-seq (clinical-grade assay)

**Summary of published cfMeDIP-seq studies evaluating circulating DNA methylation profiling for cancer detection, tumor classification, tissue-of-origin inference, treatment-response monitoring, minimal residual disease assessment, and prognostic stratification across different cancer types.**

*ALK* anaplastic lymphoma kinase, *AML* acute myeloid leukemia, *AUROC* area under the receiver operating characteristic curve, *BC* breast cancer, *cfDNA* cell-free DNA, *CRC* colorectal cancer, *CRPC* castration-resistant prostate cancer, *ctDNA* circulating tumor DNA, *DMRs* differentially methylated regions, *ER* estrogen receptor, *GC* gastric cancer, *GEO* Gene Expression Omnibus, *HGSOC* high-grade serous ovarian cancer, *HNSCC* head and neck squamous cell carcinoma, *HPV* human papillomavirus, *HR* hazard ratio, *ICB* immune checkpoint blockade, *LC* lung cancer, *LFS* Li-Fraumeni syndrome, *MEL* melanoma, *MRD* minimal residual disease, *mRCC* metastatic renal cell carcinoma, *MST* mixed solid tumors, *NACT* neoadjuvant chemotherapy, *NE* neuroendocrine, *NEPC* neuroendocrine prostate cancer, *NSCLC* non-small cell lung cancer, *OC* ovarian cancer, OS overall survival, *pCR* pathological complete response, *PDAC* pancreatic ductal adenocarcinoma, *PFS* progression-free survival, *PRAD* prostate adenocarcinoma, *PRIME* peripheral blood leukocyte methylation subtraction algorithm, *RCC* renal cell carcinoma, *RD* residual disease, *RFS* recurrence-free survival, *SCLC* small-cell lung cancer, *TCGA* The Cancer Genome Atlas, *TNBC* triple-negative breast cancer, *UBC* urothelial bladder cancer, *UM* uveal melanoma, *WGBS* whole-genome bisulfite sequencing.

### Tumor classification and tissue-of-origin inference

DNA methylation landscapes are remarkably tissue-type-specific and epigenetically stable, providing a molecular memory that persists through cell divisions and across physiological states. This intrinsic stability underlies TOO inference and is based on the assumption that cfDNA retains tissue-specific methylation signatures that can be deconvolved through comparison with reference methylation atlases. In a landmark study, Moss et al. demonstrated that the methylation fingerprint of plasma cfDNA mirrors its cellular source, showing that in healthy individuals, most cfDNA originates from immune and erythroid progenitor cells, whereas in cancer patients (colon, lung, breast, prostate), tumor-derived cfDNA contributes distinct, tissue-specific methylation signatures^42^. These findings established the conceptual basis for methylome-based liquid biopsies, later realized through cfMeDIP-seq.

The cfMeDIP-seq method was first introduced by Shen et al., who optimized a bisulfite-free, immunoprecipitation-based workflow capable of profiling genome-wide methylomes from as little as 1–10 ng of plasma cfDNA^15,16^. In a discovery cohort of 189 samples encompassing seven types, including pancreatic, colorectal, breast, lung, renal, and bladder cancers, acute myeloid leukemia, and healthy controls, the authors identified more than 14,000 DMRs in plasma that closely mirrored tumor-tissue methylation profiles (*P* < 10⁻⁴⁷). Machine-learning classifiers trained on cfMeDIP-seq data achieved area under the receiver operating characteristic curve (AUROC) values of 0.92–0.98 across cancer types, with comparable accuracy in early- and late-stage disease, demonstrating the ability to classify malignancies and infer TOO using genome-wide methylation features. Importantly, these approaches rely on supervised learning frameworks trained on reference datasets, highlighting the critical role of atlas quality and cohort composition in model performancee^15,16^. Building on this proof of principle, large-scale analyses involving more than 1000 plasma samples spanning 20 solid tumors and healthy donors confirmed the reproducibility and scalability of cfMeDIP-seq^43,44^. Using Random Forest and ElasticNet models trained on over 25,000 DMRs, these studies achieved AUROC ≈ 0.99 for distinguishing cancer from controls and 94% accuracy in TOO classification^45^. Shared hypermethylation signatures were observed across epithelial tumors in HOX, SOX, GATA, and FOX gene families, while organ-specific DMRs enabled precise tumor stratification. This validation demonstrated the robustness and transferability of cfMeDIP-seq methylation fingerprints, underscoring their potential for population-level screening and pan-cancer classification.

More recently, large-scale pancancer integration efforts have further strengthened this framework by harmonizing cfMeDIP-seq datasets across multiple studies and cancer types^44^. In a compendium of 1294 plasma cfDNA methylomes spanning 11 tumor types, uniformly processed through a standardized computational pipeline to minimize technical and biological confounders, over 14,000 recurrent pancancer DMRs were identified, alongside cancer-specific signatures enabling subtype discrimination. Importantly, integration of methylome features with cfDNA fragmentomic profiles, including fragment size distribution, nucleosome positioning, and 5′ end motifs, significantly improved cancer detection and classification performance, with machine-learning models achieving near-perfect discrimination in training datasets and robust validation in independent cohorts^44^. These findings highlight the importance of data harmonization, large-scale reference compendia, and multi-feature integration in enhancing the accuracy, generalizability, and clinical scalability of cfMeDIP-seq-based tumor classification frameworks^44^.

The performance of TOO inference is strongly influenced by tumor fraction and disease stage. Higher accuracy is generally observed in advanced cancers, where tumor-derived cfDNA is more abundant, whereas sensitivity may be reduced in early-stage disease or minimal residual disease (MRD) settings due to a lower signal-to-noise ratio. In addition, biological variability, tumor heterogeneity, and incomplete or non-representative reference methylation atlases can limit the robustness of classification models, particularly when distinguishing closely related tissues or tumors with overlapping epigenetic profiles^44^.

In parallel, cfMeDIP-seq was extended into a multi-epigenomic framework that integrates DNA methylation immunoprecipitation with histone-mark profiling (H3K4me3, H3K27ac, Pan-H3Ac)^43^. While conceptually distinct, these technologies interrogate different layers of epigenetic regulation. Across 433 patients with 15 tumor types, this approach inferred promoter and enhancer activity from 1 mL of plasma, correlating histone-associated cfDNA signals with gene-expression programs (*ρ* = 0.95; *p* < 2 × 10⁻¹⁶)^43^. It accurately predicted molecular subtypes and receptor status, such as estrogen receptor (ER)/progesterone receptor (PR) expression in breast cancer (BC) (AUC 0.91–0.94) and androgen receptor (AR)-enhancer activation in prostate cancer (PC), and captured transcriptional dependencies including HIF-2α activity in renal carcinoma and neuroendocrine lineage reprogramming across tumor types^43^. These findings demonstrate that cfMeDIP-seq-derived methylation data can be expanded to infer chromatin activity and gene-regulatory networks directly from plasma.

Collectively, these studies show that cfMeDIP-seq provides a scalable, high-resolution platform for tumor classification and TOO inference, although its performance depends on tumor fraction, reference datasets, and model design, and its application in early detection and low tumor-burden settings remains an area of active investigation.

### Cancer detection

The ability to detect malignant disease at an early, potentially curable stage remains one of the central goals of liquid-biopsy research. By capturing methylation signatures from low amounts of cfDNA, cfMeDIP-seq has emerged as a powerful tool for non-invasive early detection across a broad range of solid tumors. Unlike mutation-based assays, which depend on high tumor fraction and recurrent driver alterations, cfMeDIP-seq profiles epigenomic aberrations that are both abundant, tissue-, and cancer-specific, providing a biologically stable readout of tumor identity even when ctDNA levels are exceedingly low.

#### Renal and urothelial cancers

The first independent clinical validation of cfMeDIP-seq was reported by Nuzzo et al., analyzing 148 plasma and urine samples from patients with renal cell carcinoma (RCC) and urothelial carcinoma (UC) across all stages, including approximately one-third with stage I/II disease^20^. Plasma-based classification of RCC versus controls achieved an AUROC of 0.99, while discrimination between RCC and UC reached 0.979. Notably, methylation scores showed no correlation with stage or histology, indicating robust detection even in early-stage tumors^20^. Urine cfDNA profiling yielded an AUROC of 0.858, representing the first demonstration of cfMeDIP-seq feasibility in urine and underscoring its potential for screening through non-blood biofluids^20^. In a direct comparison of epigenetic and mutational assays, cfMeDIP-seq was evaluated against targeted variant sequencing in 40 patients with metastatic RCC^21^. Whereas variant analysis identified mutations in only 28% of cases, often confounded by clonal hematopoiesis, cfMeDIP-seq correctly classified 100% of RCC samples (AUROC: 0.983; specificity: 88%)^21^. These results highlight the high sensitivity of methylome-based cfDNA profiling in cancers with low mutational burden and minimal ctDNA shedding, establishing cfMeDIP-seq as a highly sensitive platform for early RCC detection.

#### Prostate cancer

Among genitourinary malignancies, PC represents one of the most extensively studied models for cfDNA methylome analysis. Genome-wide cfMeDIP-seq profiling has revealed that circulating methylation signatures not only enable sensitive disease detection but also mirror the biological continuum of tumor progression and lineage plasticity. Comprehensive methylome mapping across 235 plasma samples encompassing localized and metastatic disease provided the first global view of cfDNA methylation in PC^19^. The analysis revealed pervasive promoter hypermethylation of tumor-suppressor genes, particularly within CpG islands, accompanied by extensive hypomethylation of pericentromeric repetitive elements linked to chromosomal instability. Among the DMRs, the glucocorticoid-receptor gene (NR3C1) displayed a context-dependent pattern: in localized tumors, promoter hypermethylation correlated with down-regulation of immune-related transcriptional programs and poorer outcomes, whereas in metastatic disease, the same modification exhibited an inverse prognostic association^19^. This apparent epigenetic “switch” suggests dynamic adaptation of glucocorticoid-receptor signaling during progression, possibly reflecting cumulative corticosteroid exposure or microenvironmental influence. A Random-Forest model trained on cfMeDIP-seq data achieved an AUC of 0.989 for discriminating localized from metastatic samples, maintaining high accuracy even at low tumor fractions^19^.

Epigenetic plasticity also plays a critical role in the evolution of treatment-resistant phenotypes such as neuroendocrine prostate cancer (NEPC). A tissue-informed cfDNA methylation framework developed to detect this aggressive variant leveraged MeDIP-seq data from patient-derived xenografts to define NEPC- and adenocarcinoma-enriched DMRs^46^. Integration of these signatures into a plasma-based NEPC risk score yielded AUROC values of 0.96–1.0 with 100% sensitivity and up to 95% specificity across independent cohorts^46^. High scores were associated with shorter overall survival (OS), and several patients classified as high-risk harbored *RB1* or *TP53* loss or showed rapid clinical progression despite displaying adenocarcinoma histology^46^. These results demonstrate that cfMeDIP-seq can sensitively detect lineage plasticity and emerging NE differentiation, providing an early warning signal for therapeutic resistance.

#### Ovarian cancer

cfMeDIP-seq has also proven valuable for the non-invasive detection and molecular characterization of epithelial ovarian cancer. In a prospective, multi-institutional study, plasma methylome profiling achieved an AUROC of 0.96 for distinguishing high-grade serous ovarian carcinoma (HGSOC) from healthy controls and 0.93 for distinguishing HGSOC from benign lesions, with values that significantly surpassed the performance of the serum marker CA-125^47^. Tumor-specific hypermethylation was concentrated at developmental transcription factors, including HOXA9, PAX8, and SOX17, loci crucial for Müllerian differentiation and ovarian epithelial lineage identity^47^. Remarkably, these alterations were detectable up to a year before clinical diagnosis, suggesting the potential of cfMeDIP-seq for early screening and risk stratification. Genome-wide profiling in an independent cohort further confirmed the breadth and biological coherence of these findings^48^. Across 536 DMRs, 97% showed hypermethylation in cfDNA from HGSOC compared with benign and healthy controls^48^. Consistent changes in TBX3, HOXD3, ZFAT, and EXT1 were enriched in pathways regulating embryonic morphogenesis, endocrine signaling, and extracellular-matrix remodeling, hallmarks of serous-ovarian-cancer pathogenesis and chemoresistance^48^.

#### Breast cancer

cfMeDIP-seq has recently shown strong potential for both molecular stratification and integrative cfDNA biology in BC. In a study exploring hereditary contexts, cfMeDIP-seq was applied to plasma from patients with *BRCA1/2*-mutated BC, healthy non-carriers, and unaffected *BRCA1/2*-mutated carriers to determine whether methylation profiles could distinguish mutation-associated tumors^49^. The analysis identified 7095 hypermethylated and 212 hypomethylated regions, largely concentrated in promoters and proximal regulatory domains (1–5 kb upstream of transcription-start sites). Hypermethylation predominantly affected genes involved in Rho/RAS GTPase signaling, such as *DLC1, RASSF1, and ARHGAP5*, while hypomethylated regions were enriched in DNA-repair and cell-cycle regulators, including *FANCA, RAD50, ATR*, and *ERCC4*^49^. These methylation changes mirrored BC-specific tissue signatures from TCGA-BC data, validating their biological relevance. Machine-learning models achieved an AUC of 0.95, with 88% sensitivity and 94.7% specificity, supporting the feasibility of cfMeDIP-seq for early detection and molecular subtyping in hereditary BC^49^. An optimized cfMeDIP-seq protocol incorporating molecular barcoding was subsequently developed to jointly analyze cfDNA methylation and fragmentation patterns^50^. In plasma samples from patients with BC and healthy controls, 2211 DMRs were identified, with short cfDNA fragments (100–150 bp) markedly enriched in hypomethylated regions, consistent with increased chromatin accessibility and nuclease activity in tumor cells^50^. Integration of methylation-dependent fragmentation profiles yielded an AUC value of 0.91 with 100% specificity in validation cohorts^50^. Notably, short-fragment-enriched hypomethylated regions included *TRAF3IP3, PTPRN2*, and *GALNT9*, linking demethylation-driven chromatin remodeling to distinctive cfDNA size patterns.

#### Lung cancer

cfMeDIP-seq has demonstrated high diagnostic accuracy for the early detection and classification of lung cancer, providing a methylation-based potential alternative to imaging and mutation analysis. In a prospective study of patients with indeterminate pulmonary nodules, plasma cfDNA methylome profiling achieved an AUROC of 0.96 for distinguishing malignant lesions from benign nodules and healthy controls^51^. This performance significantly exceeded that of CT-derived risk scores, underscoring the value of epigenetic biomarkers for refining diagnostic specificity in lung-cancer screening. The DMRs identified were enriched in genes regulating epithelial differentiation, immune modulation, and oncogenic signaling, reflecting both tumor-intrinsic and microenvironmental alterations detectable at early stages^51^. Beyond its discriminative power, cfMeDIP-seq revealed consistent methylation signatures across histologic subtypes, including adenocarcinoma and squamous-cell carcinoma, suggesting conserved epigenetic programs driving lung carcinogenesis^51,52^.

#### Gastrointestinal malignancies

cfMeDIP-seq has emerged as a robust tool for the non-invasive detection of gastrointestinal (GI) malignancies, enabling high-resolution methylome profiling from minimal plasma input. In gastric cancer, plasma cfDNA methylome analysis of 250 subjects identified 21 DMRs discriminating patients from controls with an AUROC of 0.94–0.96, outperforming conventional serum biomarkers (CA19-9 and CEA)^53^. The hypermethylated loci were enriched in developmental and epithelial-differentiation pathways, revealing a consistent epigenetic landscape across gastric carcinogenesis.

In colorectal cancer, cfMeDIP-seq captured promoter hypermethylation in canonical Wnt-pathway genes, including *HOXA5, SFRP2*, and *SOX17*, achieving an AUROC of approximately 0.94 for tumor versus control discrimination^54^. Collectively, these studies demonstrate that cfMeDIP-seq offers a bisulfite-free, high-fidelity alternative for early detection of GI tumors. Using concise multi-gene methylome panels, cfMeDIP-seq enables sensitive, tissue-specific cancer identification, supporting its potential application in early detection and diagnostic triage in GI oncology.

#### Brain tumors

The first application of cfMeDIP-seq to brain malignancies provided compelling evidence that plasma methylation profiling can achieve diagnostic resolution across diverse intracranial tumor types. In a landmark study involving 220 patients with gliomas, meningiomas, glioneuronal tumors, hemangiopericytomas, and brain metastases, a machine-learning classifier trained on the top 300 DMRs reached an AUROC of 0.99 for detecting gliomas, with comparable performance across IDH-mutant (0.992) and wild-type (0.993) subtypes and across WHO grades II–IV^41^. Beyond glioma detection, cfMeDIP-seq accurately distinguished among intracranial tumor classes, achieving AUCs of 0.89 for meningioma, 0.95 for hemangiopericytoma, and 0.93 for glioneuronal tumor^41^. Plasma-derived methylation profiles correlated with corresponding tissue methylomes (*r* ≈ 0.4), confirming the tumor origin of cfDNA and the biological fidelity of cfMeDIP-seq signals. This seminal work established cfMeDIP-seq as a non-invasive liquid biopsy tool for central nervous system (CNS) tumors, capable of providing molecular classification and subtype-level information from minimal plasma DNA. By enabling epigenetic diagnosis where tissue access is limited, or biopsy carries risk, cfMeDIP-seq represents a promising approach for precision neuro-oncology through circulating-methylome analysis.

### Monitoring minimal residual disease

Even after treatments that eradicate more than 99% of the tumor burden, small populations of malignant cells, termed MRD, can persist and eventually lead to relapse^55^. Conventional imaging typically detects recurrence only once tumor burden exceeds approximately 10⁶ cells, at which point therapeutic options are often limited and less effective^56^. Therefore, implementing ultrasensitive, non-invasive strategies for early MRD detection is critical for enabling timely intervention and improving long-term outcomes. Rather than relying on recurrent driver mutations, cfMeDIP-seq captures ctDNA through differential methylation signatures, molecular features that are abundant, tissue-specific, and stable even at very low cfDNA input levels, making it particularly well suited for MRD monitoring^15,16^.

#### Breast cancer

A recent study explored the potential of cfMeDIP-seq to monitor therapy response and detect MRD in early-stage BC^57^. Seven women with triple-positive disease undergoing neoadjuvant chemotherapy (NACT) were longitudinally profiled at three time points: before treatment, after NACT but prior to surgery, and post-surgery. Genome-wide cfDNA methylation analysis revealed striking differences between patients achieving pathological complete response (pCR) and those with residual disease (RD)^57^. Patients with pCR exhibited extensive methylome remodeling, with significant hypomethylation in 307 regions and hypermethylation in 151 regions after chemotherapy, followed by partial reversal after surgery^57^. In contrast, RD patients showed negligible changes across all timepoints, suggesting limited therapeutic impact at the molecular level. Pathway enrichment analysis of DMRs highlighted dynamic regulation of metabolic and immune-related processes, including lipid metabolism, xenobiotic processing, and IL2-STAT5 signaling, indicating systemic host–tumor interactions in responders. However, the biological interpretation of these methylation changes remains limited, as cfMeDIP-seq captures signals derived from both tumor and non-tumor compartments, including immune cells, which are known to be affected by chemotherapy. As a result, it is not possible to clearly distinguish tumor-specific effects from systemic treatment-related changes. Principal component analysis demonstrated clear separation between pCR and RD groups, underscoring cfMeDIP-seq’s potential to non-invasively distinguish response patterns and identify residual disease before clinical relapse^57^. Importantly, these findings should be interpreted with caution due to several limitations, including the very small sample size, the lack of independent validation, and the absence of direct comparison with established MRD biomarkers such as mutation-based ctDNA assays. In addition, the observational nature of the study and the heterogeneity of cfDNA sources limit the immediate clinical applicability of the results.

#### Head and neck cancer

Further validation of cfMeDIP-seq for MRD detection was provided by a multicenter study involving 325 patients with head and neck cancer treated with curative intent^58^. A tissue-agnostic, genome-wide methylation enrichment assay was used to monitor cfDNA longitudinally, independent of HPV status or tumor site. cfMeDIP-seq achieved a sensitivity of 91% and a specificity of 88% for recurrence detection, with MRD positivity strongly correlating with adverse outcomes, hazard ratios of 35.7 [95% confidence interval (CI), 10.8–117.8; *P* < 0.0001] for recurrence-free survival and 47.0 [95% CI, 6.2–356.0; *P* < 0.0001] for OS^58^. Importantly, cfMeDIP-seq identified molecular relapse a mean of 4.1 months before clinical recurrence, providing a substantial lead time for therapeutic intervention^58^. These associations remained significant across HPV-positive and HPV-negative subgroups, and multivariate models confirmed MRD status as an independent prognostic factor, outperforming conventional clinicopathologic variables^58^. However, several limitations should be noted. The retrospective, single-center design may limit generalizability, and predefined sampling timepoints could underestimate assay sensitivity. Moreover, the tissue-agnostic approach may capture non-tumor signals, and the lack of comparison with established ctDNA-based MRD assays, together with the need for prospective validation, limits immediate clinical applicability.

### Response prediction and treatment monitoring

The capacity to predict therapeutic response and monitor disease dynamics in real time represents one of the most promising clinical applications of cfMeDIP-seq. By quantifying methylation-based tumor signals in cfDNA, cfMeDIP-seq provides a molecular window into treatment efficacy, enabling early identification of responders, detection of emerging resistance, and longitudinal mapping of tumor evolution.

In metastatic PC, cfDNA methylome profiling revealed that therapy-induced methylation remodeling accurately mirrored response to androgen receptor signaling inhibitors^59^. Responders to enzalutamide or abiraterone displayed coordinated demethylation of AR-regulated loci and metabolic genes, whereas non-responders maintained stable hypermethylation at NKX3-1, KLK3, and FASN, indicating transcriptional silencing associated with therapeutic resistance^59^. A cfMeDIP-seq-derived response score achieved an AUROC of 0.91, outperforming PSA kinetics and mutational load, demonstrating the ability of cfDNA methylation dynamics to forecast treatment resistance before radiographic or biochemical evidence of progression^59^. These observations position cfMeDIP-seq as a functional readout of AR pathway activity, capturing epigenetic states associated with therapeutic sensitivity and resistance. However, the mechanistic attribution of these signals remains uncertain, as cfDNA methylation integrates tumor and host-derived contributions. Moreover, the limited cohort size and lack of external validation constrain the robustness and generalizability of these findings, underscoring the need for prospective and standardized evaluation.

Beyond baseline stratification, cfMeDIP-seq enables longitudinal tracking of epigenetic remodeling under therapy, providing insights into molecular relapse, adaptive resistance, and tumor evolution. In ALK-rearranged non-small cell lung cancer (NSCLC), cfMeDIP-seq applied to serial plasma samples from patients receiving tyrosine-kinase inhibitors identified 245 ALK-specific DMRs enriched in genes silenced in lung adenocarcinoma (PCDH10, TBX2, CDO1, HOXA9)^18^. The composite 5mC score correlated strongly with EML4-ALK fusion abundance (*ρ* > 0.6) and chromosomal instability, serving as a surrogate for tumor burden. Rising methylation scores anticipated radiologic progression by a median of 3 months, demonstrating cfMeDIP-seq’s ability to detect molecular relapse in ALK-positive NSCLC even when mutation-based assays were non-informative^18^.

Consistent with these findings, the cfMeDIP-seq approach was shown to robustly track treatment response and residual disease dynamics in HPV-negative head and neck squamous cell carcinoma^60^. In locoregionally confined disease, cfMeDIP-seq was applied in a multimodal plasma-profiling framework alongside mutation analysis in cancer patients and risk-matched controls. Differential methylation analysis identified ctDNA-derived hypermethylated regions enriched for CpG islands and disease-specific patterns, with methylation-based ctDNA abundance strongly correlating with mutation-based ctDNA^60^. Importantly, patients with detectable ctDNA by both modalities exhibited significantly worse OS independent of clinical stage, and failure to clear ctDNA after curative therapy correlated with recurrence, reinforcing the utility of cfMeDIP-seq for response monitoring and early molecular relapse detection.

Complementing these findings, an integrated cfMeDIP-seq and fragmentomic analysis in a multi-cancer cohort, including NSCLC patients treated with pembrolizumab, evaluated cfDNA dynamics under PD-1 blockade^45^. Responders exhibited early demethylation of interferon-response and immune-activation loci, correlating with enhanced T-cell transcriptional programs, whereas non-responders retained hypermethylation of immune-exhaustion genes and displayed increasing methylation entropy, reflecting clonal diversification and immune escape^45^. The composite methylation-fragmentation score correlated strongly with ctDNA abundance (Spearman *ρ* > 0.6, *p* < 0.015) and predicted OS independently of PD-L1 expression and tumor-mutation burden, confirming cfMeDIP-seq’s potential to resolve immune-modulatory responses and emerging resistance during checkpoint inhibition^45^.

Recently, a clinical-grade, tissue-agnostic cfMeDIP-seq platform has been prospectively validated for response monitoring to immune checkpoint blockade across multiple solid tumors^61^. In a phase II study evaluating pembrolizumab, early decreases in ctDNA methylation scores from baseline to cycle 3 were strongly associated with OR clinical benefit, and significantly improved PFS, whereas no patients with increasing ctDNA achieved OR^61^. Importantly, ctDNA dynamics provided independent and more consistent predictive value compared with PD-L1 expression and tumor mutational burden, supporting the role of cfMeDIP-seq as a minimally invasive, real-time biomarker of immunotherapy efficacy and early resistance^61^. These findings extend the utility of cfDNA methylation profiling beyond disease detection, positioning it as a clinically actionable tool for early treatment adaptation in heterogeneous cancer populations.

Finally, cfMeDIP-seq has been applied to predict organ-specific metastatic progression. In patients with lung adenocarcinoma, a 5553-CpG methylation signature was identified that predicted CNS tropism, achieving an AUROC of 0.81 for brain-metastasis development within 5 years^62^. Methylation changes affecting neural-lineage and extracellular-matrix-related genes distinguished CNS progression from systemic dissemination, revealing epigenetic adaptations associated with metastatic organotropism and highlighting potential biomarkers of metastatic spread^62^.

## Challenges and limitations

The clinical value of liquid biopsy is increasingly recognized, and sample collection remains one of its most critical determinants. Blood is the most widely used material in research and clinical settings due to its minimally invasive nature and the wealth of biological information it provides. However, cfDNA-based studies face significant pre-analytical and technical challenges that can affect cfMeDIP-seq reproducibility and interpretability.

In clinical practice, blood sampling is often performed during routine management and must be followed by rapid processing to avoid leukocyte lysis and genomic-DNA contamination^63^. Plasma is the preferred matrix because serum exhibits higher nuclease activity and sustained cfDNA degradation^64,65^. EDTA-plasma ensures better cfDNA stability, provided that samples are kept on ice and processed within 2 h of collection^66,67^. Time and temperature are critical: cfDNA has a short half-life (16 min–2.5 h), and delays rapidly increase wild-type background^68^. Studies comparing collection tubes have shown that K₂/K₃EDTA preserve cfDNA integrity more reliably than serum or Streck tubes, whereas PAXgene tubes maintain methylation fidelity and allow short-term transport at 4 °C^69,70^. These pre-analytical variables represent major sources of variability across laboratories rather than standardized procedural steps. Standardized plasma isolation and storage are essential. Dual centrifugation, typically 800–1200 × *g* followed by 14,000–16,000 × *g* at 4 °C, minimizes residual debris, and plasma should be stored at −80 °C in small aliquots to prevent degradation^71^. Table 3 summarizes the key pre-analytical and analytical parameters, such as collection tube type, processing time, centrifugation protocol, and cfDNA isolation kit, used in studies applying cfMeDIP-seq. A marked heterogeneity in processing, centrifugation speeds, and DNA extraction methods, with only 44% performing dual spins and most relying on manual Qiagen kits, can be observed. Such variability highlights the urgent need for harmonized pre-analytical workflows, as even minor deviations can influence cfDNA yield, fragment distribution, and methylation-enrichment efficiency^72^. Given cfMeDIP-seq’s sensitivity to fragment integrity and CpG density, standardized handling protocols are a prerequisite for reliable clinical translation. These sources of variability can significantly affect signal-to-noise ratio and limit inter-study comparability.

**Table 3:** Pre-analytical and analytical parameters across published cfMeDIP-seq studies

Study/Year	Cancer type(s)	Blood collection tube	Matrix	Processing time	Centrifugation protocol	Plasma storage conditions	Plasma volume for cfMeDIP_seq	cfDNA extraction kit	cfDNA quantity for cfMeDIP_seq
**Cancer detection/early detection**									
Shen et al., Nature 2018	Pancreatic ductal adenocarcinoma (PDAC), lung cancer (LC), breast cancer (BC), colorectal cancer (CRC), renal cell carcinoma (RCC), urothelial bladder cancer (UBC), and acute myeloid leukemia (AML), plus healthy controls	EDTA and ACD	Plasma	NA	NA	−80 °C	0.5–3.5 mL	QIAamp Circulating NA Kit (Qiagen, cat. no. 55114)	10 ng
Nuzzo et al., Nat Med 2020	Renal cell carcinoma (RCC); urothelial bladder cancer (UBC); controls	EDTA	Plasma	NA	Dual (2500 × *g* at r.t. for 10 min, 2500 × *g* at r.t. for 10 min)	−80 °C	1 mL	QIAamp Circulating NA Kit (Qiagen, cat. no. 55114)	NA
Nassiri et al., Nat Med 2020	Brain tumors (glioma, meningioma, hemangiopericytoma, brain metastases)	ACD	Plasma	≤0.5 h	Single (2500 × *g* at 20 °C for 15 min)	NA	NA	QIAamp Circulating NA Kit (Qiagen, cat. no. 55114)	1–10 ng
Lasseter et al., Genet Med 2020	Metastatic renal cell carcinoma (mRCC)	EDTA	Plasma	≤1 h	Dual (2000 × *g* at 4 °C for 15 min, 3000 x *g* for 20 min)	−80 °C	2–10 mL	QIAamp Circulating NA Kit (Qiagen, cat. no. 55114)	1.64–10 ng
Qi et al., Cancer Science 2021	Lung cancer (LC)	EDTA	Plasma	≤4 h	Dual (1600 × *g* at r.t. for 10 min, 16,000 × *g* at r.t. for 10 min)	−80 °C	3–4 mL	QIAamp Circulating NA Kit (Qiagen, cat. no. 55114)	NA
Lu et al., Clin Epigenetics 2022	Ovarian cancer	EDTA	Plasma	≤48 h	NA	−80 °C	2–4 mL	NA	10 ng
Zhang et al., World J Surg Oncol 2022	Colorectal cancer (CRC)	EDTA	Plasma	NA	Dual (1900 × *g* at 4 °C for 10 min; 16,000 × *g* at 4 °C for 10 min)	−80 °C	NA	QIAamp Circulating NA Kit (Qiagen, cat. no. 55114)	~50 ng
Wang, J., et al., Epi & Chromatin 2023	Breast cancer	EDTA	Plasma	NA	Dual (1000 × *g* at 4 °C for 10 min, 16,000 × *g* at 4 °C for 10 min)	−80 °C	NA	MiniMaxTM High Efficiency Cell-Free DNA Isolation Kit (Apostle, cat. no. A17622-250)	~10 to 20 ng
Qi et al., Cancer Science 2024	Gastric cancer (GC)	EDTA	Plasma	≤4 h	Dual (1600 × *g* for 10 min; 16,000 × *g* at 4 °C for 10 min)	−80 °C	4 mL	QIAamp Circulating NA Kit (Qiagen, cat. no. 55114)	NA
Terp et al., Cancers 2025	Ovarian cancer (OC); benign ovarian conditions; healthy controls	EDTA	Plasma	≤2 h	Single (2000 × *g* for 10 min at 18 °C using a slow brake)	−80 °C	1–2.1 mL	QIAamp Circulating NA Kit (Qiagen, cat. no. 55114)	NA
Zeng et al., Nature Cancer 2026	Brain cancer, lung cancer (LC), prostate cancer (PC), acute myeloid leukemia (AML), pancreatic ductal adenocarcinoma (PDAC), uveal melanoma (UM), head and neck squamous cell carcinoma (HNSCC), breast cancer (BC), colorectal cancer (CRC), urothelial bladder cancer (UBC), renal cell carcinoma (RCC); healthy controls; Li-Fraumeni syndrome (LFS)	EDTA	Plasma	NA	NA	−80 °C	1 mL	QIAamp Circulating NA Kit (Qiagen, cat. no. 55114)	1–10 ng
**Tumor classification/tissue of origin**									
Baca et al., Nat Med 2023	Advanced solid tumors (breast, lung, prostate, esophageal, Merkel cell carcinoma, melanoma, glioma)	K₂EDTA	Plasma	≤6 h	Dual (1500 × *g* at 4 °C for 10 min; 1500 × *g* at 4 °C for 10 min).	−80 °C	NA	QIAamp Circulating NA Kit (Qiagen, cat. no. 55114)	NA
Berchuck et al., Clin Cancer Res. 2022	Neuroendocrine prostate cancer (NEPC) vs CRPC adenocarcinoma (PRAD)	EDTA	Plasma	NA	Dual (2500 × *g* at r.t. for 10 min, 2500 × *g* at r.t. for 10 min)	−80 °C	1 mL	QIAamp Circulating NA Kit (Qiagen, cat. no. 55114)	NA
Chen et al., Nat Commun 2022	Prostate cancer (localized and metastatic) Tumor classification/cancer detection	EDTA	Plasma	NA	NA	−80 °C	500 µl–1 ml	QIAamp Circulating NA Kit (Qiagen, cat. no. 55114)	5 ng
Zuccato et al., Nat Med 2023	Brain metastases	ACD	Plasma	NA	Single (2500 × *g* at 20 °C for 15 min)	NA	0.5–3 mL	QIAamp Circulating NA Kit (Qiagen, cat. no. 55114)	1–10 ng
Haq et al., iScience 2022	Small-cell lung cancer (SCLC)	EDTA	Plasma	NA	Dual (4000 × *g* at 4 °C for 10 min, 16,000 × *g* at 4 °C for 10 min)	−80 °C	NA	QIAamp Circulating NA Kit (Qiagen, cat. no. 55114)	10 ng
Grisolia et al., Clin Epigenetics 2025	Breast cancer (BRCA1/2mut)	EDTA	Plasma	NA	Dual (1200 × *g* for 20 min, 21,300 × *g* for 20 min)	−80 °C	1 mL	QIAamp Circulating NA Kit (Qiagen, cat. no. 55114)	NA
**Minimal residual disease/treatment monitoring**									
Peter et al., Epigenomics 2020	Metastatic castration-resistant prostate cancer (mCRPC)	BD Vacutainer® CPT™ (Bioscience, cat. no. 362760)	Plasma	≤2 h	Dual (1800 × *g* for 20 min; 16,000 × *g* at 37 °C for 5 min)	−80 °C	~4 mL	QIAamp Circulating NA Kit (Qiagen, cat. no. 55114)	NA
Burgener et al., Clin Cancer Res 2021	HPV-negative head and neck squamous cell carcinoma (HNSCC)	EDTA	Plasma	≤1 h	NA	−80 °C	NA	QIAamp Circulating NA Kit (Qiagen, cat. no. 55114)	10–20 ng
Janke et al., Clin Epigenetics 2022	ALK-positive non-small-cell lung cancer (NSCLC)	K₂EDTA	Plasma	≤1 h	Dual (1600 × *g* for 10 min without brake, 3400 × *g* for 10 min without brake)	−80 °C	2 mL	QIAamp MinElute ccfDNA Kit (Qiagen, cat. no. 55284)	NA
Ravera et al., The journal of Liquid biopsy 2024	Breast cancer (early, triple-positive)	EDTA	Plasma	NA	NA	NA	~4 mL	QIAamp MinElute ccfDNA Kit (Qiagen, cat. No. 55284)	NA
Liu et al., Ann. Oncol. 2024	Head and neck cancer (HNSCC, HPV+ and HPV−)	EDTA	Plasma	≤2 h	NA	−80 °C	NA	NA	
Zhao et al., Cancer Discov 2024	Multiple solid tumors (TNBC, HGSOC, HNSCC, melanoma, others)	EDTA	Plasma	≤2 h	NA	−80 °C	NA	QIAamp Circulating NA Kit (Qiagen, cat. no. 55114)	10 ng
Stutheit-Zhao et al., npj Precision Oncology 2026	Head and neck squamous cell carcinoma (HNSCC), triple-negative breast cancer (TNBC), high-grade serous ovarian cancer (HGSOC), melanoma (MEL), mixed solid tumors (MSTs)	EDTA	Plasma	≤2 h	NA	−80 °C	1 mL	QIAamp Circulating NA Kit (Qiagen, cat. no. 55114)	5–10 ng

**Summary of blood collection, plasma processing, and cfDNA isolation workflows used in cfMeDIP-seq studies across different cancer types.**

*cfMeDIP-seq* circulating cell-free methylated DNA immunoprecipitation sequencing, *cfDNA* circulating cell-free DNA, *EDTA* ethylenediaminetetraacetic acid, *K₂EDTA* dipotassium ethylenediaminetetraacetic acid, *ACD* acid citrate dextrose, *CPT* cell preparation tube, *BD* Becton Dickinson, *r.t.* room temperature, *g* relative centrifugal force, *°C* degrees Celsius, *ng* nanograms, *mL* milliliters, *µL* microliters, *NA* not available, *HPV* human papillomavirus, *HNSCC* head and neck squamous cell carcinoma, *TNBC* triple-negative breast cancer, *HGSOC* high-grade serous ovarian cancer, *MEL* melanoma, *MSTs* mixed solid tumors, *PDAC* pancreatic ductal adenocarcinoma, *LC* lung cancer, *BC* breast cancer, *CRC* colorectal cancer, *RCC* renal cell carcinoma, *UBC* urothelial bladder cancer, *AML* acute myeloid leukemia, *GC* gastric cancer, *OC* ovarian cancer, *NEPC* neuroendocrine prostate cancer, *CRPC* castration-resistant prostate cancer, *PRAD* prostate adenocarcinoma, *SCLC* small-cell lung cancer, *NSCLC* non-small cell lung cancer, *ALK* anaplastic lymphoma kinase, *mRCC* metastatic renal cell carcinoma, *mCRPC* metastatic castration-resistant prostate cancer, *LFS* Li-Fraumeni syndrome.

At the analytical level, cfMeDIP-seq presents specific technical and biological limitations. First, it provides regional methylation resolution (~100 bp), rather than single-CpG precision, limiting the ability to distinguish individual CpG sites^15,16^. Furthermore, the assay selectively enriches 5mC and does not directly capture 5hmC, which arises from active DNA demethylation processes and is established as a stable epigenetic mark associated with transcriptional activation. As a result, while cfMeDIP-seq effectively reflects gene silencing, it cannot directly capture active tumor gene expression profiles. This blind spot restricts the ability to identify certain clinically and prognostically relevant signals. For instance, elevated cell-free 5hmC levels in genes such as *EZH2* and *TOP2A* have been associated with aggressive PC variants and poor OS^73^.

Furthermore, the absence of signal may also reflect technical issues such as DNA fragments being lost during sample processing, suboptimal antibody binding, or insufficient sequencing depth. Although the use of input controls and spike-in normalization strategies can partially mitigate these limitations and enable quantification of methylation enrichment, they do not provide a direct measurement of unmethylated DNA or absolute methylation levels.

Biological variability adds further complexity. Although cfDNA originates from multiple tissues, more than 90% of the total cfDNA pool is of hematopoietic origin, rendering its levels susceptible to physiological processes such as aging, systemic inflammation, or metabolic shifts. cfDNA originates from multiple tissues, and methylation can be affected by aging, inflammation, or metabolic state^74–77^. These factors may introduce background methylation signals that partially overlap with tumor-associated patterns, thereby reducing specificity and complicating biological interpretation. Without comprehensive, population-diverse reference atlases, tumor-specific signals may be obscured by non-malignant background. These factors may also affect TOO inference, particularly in low-tumor-fraction settings, where background cfDNA from non-malignant tissues can obscure tumor-specific methylation signals. In addition, variability in tumor fraction can significantly influence assay sensitivity, as low levels of tumor-derived cfDNA, particularly in early-stage disease or MRD settings, may dilute methylation signals and increase the risk of false-negative results. Integration with complementary cfDNA modalities such as fragmentomics or chromatin-mark profiling could improve specificity and functional insight.

The evolution of cfMeDIP-seq into an established clinical tool requires validation in large multicenter studies, including cost-effectiveness analyses, and the implementation of automated wet-lab and computational workflows. Although more affordable than WGBS, it still has turnaround time and data-processing demands that limit scalability. A major barrier remains the lack of standardized pre-analytical and analytical frameworks, which affects inter-laboratory reproducibility. In particular, key experimental and analytical parameters remain protocol-dependent, including filler DNA composition, sequencing depth, and quality-control thresholds^78^.

## Clinical translation and future perspectives

The rapid evolution of cfMeDIP-seq from a discovery assay to a clinically informative technology epitomizes the transformation of precision oncology, where liquid biopsy is gradually aiding early detection, disease monitoring, and therapeutic decision-making in cancer^79–81^. As evidence expands, cfMeDIP-seq is progressing from proof-of-principle studies toward clinical translation, requiring coordinated efforts in analytical validation, workflow standardization, and regulatory alignment.

Although most cfMeDIP-seq applications have focused on plasma, the same enrichment protocol is adaptable to other matrices containing fragmented, low-input DNA. Feasibility has been demonstrated in urine, enabling accurate detection of renal cancer, and in cerebrospinal fluid, where cfDNA methylome profiling non-invasively classified malignant brain tumors with high concordance with matched tissue methylomes^40^. These results support its extension to biofluids such as saliva, pleural effusions, and ascitic fluid, where proximity to the tumor microenvironment may further enhance diagnostic yield.

Building on these advances, cfMeDIP-seq is entering prospective observational and multicenter evaluation across multiple clinical applications^61,82,83^. Recent studies have further strengthened the clinical evidence supporting cfMeDIP-seq-based approaches, demonstrating their applicability across different clinical contexts^44^.

Among emerging platforms, the Adela approach, which leverages genome-wide methylome enrichment coupled with machine-learning integration, is currently undergoing clinical validation in studies assessing early cancer detection, MRD monitoring, and treatment response evaluation^61,82,83^. These include large case-control cohorts with longitudinal sampling, as well as recent prospective analyses demonstrating that early changes in methylated ctDNA during immunotherapy are strongly associated with objective response, clinical benefit, and progression-free survival across multiple solid tumor types, supporting the feasibility of real-time, tissue-agnostic response monitoring in clinical practice^61^. Independent investigations have reported consistent results, reinforcing the robustness of methylation-based cfDNA profiling in capturing clinically relevant tumor dynamics across different malignancies and therapeutic contexts^44^. Moreover, exploratory data further support its potential for early detection of recurrence, with lead times preceding standard-of-care imaging in selected tumor types.

The Adela platform is also being evaluated in an ongoing large-scale case-control study (ClinicalTrials.gov Identifier: NCT05366881), which aims to enroll approximately 7000 participants to validate methylation-based signatures across multiple solid tumors and assess reproducibility and diagnostic performance^61^. Collectively, these efforts underscore the broad translational potential of cfMeDIP-seq-based approaches across the cancer continuum. However, the current evidence base is predominantly observational or retrospective, and prospective demonstration of clinical utility is still evolving. These studies are expected to inform future regulatory validation and clinical implementation of methylome-based liquid biopsy assays.

Despite increasing adoption, cfMeDIP-seq remains mainly research-focused because clinical translation demands the harmonization of pre-analytical and analytical factors, such as blood collection, plasma processing, cfDNA extraction, antibody performance, and computational pipelines. Harmonized protocols and inter-laboratory quality controls are critical to ensure reproducibility. Finally, methodological refinements, such as the use of spike-in controls, UMIs, and quantitative quality-control metrics, further mitigate batch effects and improve data normalization.

Beyond these technical optimizations, successful clinical translation requires alignment with established regulatory frameworks governing diagnostic assays, such as Clinical Laboratory Improvement Amendments and Food and Drug Administration oversight in the United States, In Vitro Diagnostic Regulation (IVDR 2017/746) in Europe, Health Canada, the Pharmaceuticals and Medical Devices Agency in Japan, and the Therapeutic Goods Administration in Australia. Across all these settings, approval is contingent upon demonstrating analytical validity (accuracy, precision, limit of detection), clinical performance (sensitivity, specificity, predictive value, and lead time), and prospective clinical utility, particularly in terms of its impact on patient management and outcomes.

Cost-effectiveness analyses will also be critical when positioning cfMeDIP-seq against mutation-based commercial assays such as Guardant360 and FoundationOne Liquid, or methylation-based tests like Galleri and Shield. Although costs may vary depending on sequencing depth, laboratory workflows, and scale, cfMeDIP-seq generally requires a substantially lower sequencing burden than WGBS, resulting in approximately a tenfold reduction in overall cost. In practice, the overall cost of cfMeDIP-seq is typically comparable to currently available commercial cfDNA assays such as Guardant360 and FoundationOne Liquid, while providing genome-wide methylation information. Compared with WGBS, which requires high-depth genome-wide coverage and significantly higher input DNA, cfMeDIP-seq achieves informative methylation profiling at lower sequencing depth and nanogram-level input, as also summarized in Table 1.

Implementation further depends on certified quality-management systems, reagent traceability, and validated bioinformatics pipelines ensuring transparency and reproducibility.

Next-generation applications will integrate multilayered cfDNA information to enhance diagnostic sensitivity, specificity, and interpretability. Combining methylome and fragmentome features improves early detection and response prediction, as shown in pembrolizumab-treated tumors, where cfDNA methylation and fragmentation findings improved outcome prediction^45^. Multi-epigenomic profiling that couples 5mC enrichment with histone-mark detection (H3K4me3, H3K27ac) infers promoter and enhancer activity, revealing transcriptional programs directly from plasma^43^. These strategies illustrate a shift toward integrated liquid-biopsy frameworks in which epigenetic, fragmentomic, and mutational signals are jointly leveraged for clinical decision-making. At the computational level, deep-learning classifiers trained on large, population-diverse cfMeDIP-seq datasets can identify subtle tumor-specific methylation patterns and predict therapy response. Comprehensive reference methylation atlases spanning populations and physiological states will be crucial for minimizing confounding and ensuring model generalization.

Looking ahead, cfMeDIP-seq is rapidly progressing toward clinical translation, although routine clinical adoption will depend on multicenter validation, regulatory harmonization, and further automation. The convergence of prospective trials, AI-assisted interpretation, and end-to-end workflows will accelerate its integration into precision oncology pipelines. Its nanogram-level input requirement, compatibility with multiple biofluids, and scalability across research and clinical contexts make cfMeDIP-seq uniquely versatile among liquid-biopsy technologies. By bridging epigenetic discovery with actionable diagnostics, cfMeDIP-seq is likely to become an important component of multi-omic precision oncology, supporting earlier detection, longitudinal surveillance, and dynamic assessment of therapeutic response within a unified, non-invasive clinical framework.

## Conclusion

cfMeDIP-seq has emerged as a robust and versatile platform for genome-wide cfDNA methylation profiling, bridging the gap between discovery-stage epigenomics and clinically actionable diagnostics. Its combination of ultra-low input requirements, high specificity, and adaptability across multiple biofluids enables sensitive detection, molecular classification, and longitudinal disease monitoring in a wide spectrum of cancers.

Ongoing prospective studies and technological advances are rapidly paving the way for clinical implementation. Achieving full translational impact will depend on standardized pre-analytical workflows, validated antibody and computational pipelines, and harmonized regulatory frameworks. As automation and multi-omic integration mature, cfMeDIP-seq is well-positioned to become an important component of next-generation liquid biopsy, supporting earlier cancer detection, dynamic response assessment, and truly personalized precision oncology.

## Data Availability

No datasets were generated or analyzed during the current study.

## References

[1] Cescon, D. W., Bratman, S. V., Chan, S. M. & Siu, L. L. Circulating tumor DNA and liquid biopsy in oncology. *Nat. Cancer* **1**, 276–290 (2020). 35122035 10.1038/s43018-020-0043-5

[2] Gao, Q. et al. Circulating cell-free DNA for cancer early detection. *Innovation* **3**, 100259 (2022). 10.1016/j.xinn.2022.100259 PMC9133648 35647572

[3] Ma, L. et al. Liquid biopsy in cancer: current status, challenges and future prospects. *Signal Transduct. Target. Ther.* **9**, 336 (2024). 39617822 10.1038/s41392-024-02021-w PMC11609310

[4] Bartolomucci, A. et al. Circulating tumor DNA to monitor treatment response in solid tumors and advance precision oncology. *npj Precis. Oncol.* **9**, 84 (2025). 40122951 10.1038/s41698-025-00876-y PMC11930993

[5] Schiavone, M. L. et al. Liquid biopsy in breast cancer: redefining precision medicine. *J. Liq. Biopsy* **9**, 100312 (2025). 40740670 10.1016/j.jlb.2025.100312 PMC12308030

[6] Bronkhorst, A. J. & Holdenrieder, S. The changing face of circulating tumor DNA (ctDNA) profiling: factors that shape the landscape of methodologies, technologies, and commercialization. *Med. Genet.* **35**, 201–235 (2023). 38835739 10.1515/medgen-2023-2065 PMC11006350

[7] Noë, M. et al. DNA methylation and gene expression as determinants of genome-wide cell-free DNA fragmentation. *Nat. Commun.* **15**, 6690 (2024). 39107309 10.1038/s41467-024-50850-8 PMC11303779

[8] Spector, B. L., Harrell, L., Sante, D., Wyckoff, G. J. & Willig, L. The methylome and cell-free DNA: current applications in medicine and pediatric disease. *Pediatr. Res.* **94**, 89–95 (2023). 36646885 10.1038/s41390-022-02448-3 PMC9842217

[9] Geissler, F. et al. The role of aberrant DNA methylation in cancer initiation and clinical impacts. *Ther. Adv. Med. Oncol.* **16**, 17588359231220511 (2024). 38293277 10.1177/17588359231220511 PMC10826407

[10] Esteller, M. Epigenetics in cancer. *N. Engl. J. Med.* **358**, 1148–1159 (2008). 18337604 10.1056/NEJMra072067

[11] Slieker, R. C. et al. Identification and systematic annotation of tissue-specific differentially methylated regions using the Illumina 450k array. *Epigenetics Chromatin* **6**, 26 (2013). 23919675 10.1186/1756-8935-6-26 PMC3750594

[12] Kundaje, A. et al. Integrative analysis of 111 reference human epigenomes. *Nature* **518**, 317–330 (2015). 25693563 10.1038/nature14248 PMC4530010

[13] Tanaka, K. & Okamoto, A. Degradation of DNA by bisulfite treatment. *Bioorg. Med. Chem. Lett.* **17**, 1912–1915 (2007). 17276678 10.1016/j.bmcl.2007.01.040

[14] de Abreu, A. R. et al. Comparison of current methods for genome-wide DNA methylation profiling. *Epigenetics Chromatin* **18**, 57 (2025). 40855329 10.1186/s13072-025-00616-3 PMC12376410

[15] Shen, S. Y., Burgener, J. M., Bratman, S. V. & De Carvalho, D. D. Preparation of cfMeDIP-seq libraries for methylome profiling of plasma cell-free DNA. *Nat. Protoc.* **14**, 2749–2780 (2019). 31471598 10.1038/s41596-019-0202-2

[16] Shen, S. Y. et al. Sensitive tumour detection and classification using plasma cell-free DNA methylomes. *Nature* **563**, 579–583 (2018). 30429608 10.1038/s41586-018-0703-0

[17] Fotia, G. et al. Outcomes of first-line abiraterone acetate or enzalutamide for older adults with metastatic castration-resistant prostate cancer according to use of upfront docetaxel for metastatic castration-sensitive prostate cancer in an international multicenter registry: a SPARTACUSS-Meet-URO 26 study. *Clin. Genitourin. Cancer* **22**, 102185 (2024). 39217072 10.1016/j.clgc.2024.102185

[18] Janke, F. et al. Longitudinal monitoring of cell-free DNA methylation in ALK-positive non-small cell lung cancer patients. *Clin. Epigenetics* **14**, 163 (2022). 36461127 10.1186/s13148-022-01387-4 PMC9719130

[19] Chen, S. et al. The cell-free DNA methylome captures distinctions between localized and metastatic prostate tumors. *Nat. Commun.* **13**, 6467 (2022). 36309516 10.1038/s41467-022-34012-2 PMC9617856

[20] Nuzzo, P. V. et al. Detection of renal cell carcinoma using plasma and urine cell-free DNA methylomes. *Nat. Med.* **26**, 1041–1043 (2020). 32572266 10.1038/s41591-020-0933-1 PMC8288043

[21] Lasseter, K. et al. Plasma cell-free DNA variant analysis compared with methylated DNA analysis in renal cell carcinoma. *Genet. Med.* **22**, 1366–1373 (2020). 32341571 10.1038/s41436-020-0801-x

[22] Lister, R. et al. Human DNA methylomes at base resolution show widespread epigenomic differences. *Nature* **462**, 315–322 (2009). 19829295 10.1038/nature08514 PMC2857523

[23] Olova, N. et al. Comparison of whole-genome bisulfite sequencing library preparation strategies identifies sources of biases affecting DNA methylation data. *Genome Biol.* **19**, 33 (2018). 29544553 10.1186/s13059-018-1408-2 PMC5856372

[24] Miura, F. et al. Highly efficient single-stranded DNA ligation technique improves low-input whole-genome bisulfite sequencing by post-bisulfite adaptor tagging. *Nucleic Acids Res.* **47**, e85–e85 (2019). 31114914 10.1093/nar/gkz435 PMC6736019

[25] Miura, F. & Ito, T. Post-bisulfite adaptor tagging for PCR-free whole-genome bisulfite sequencing. in *DNA Methylation Protocols* (ed. Tost, J.) 123–136 (Springer New York, 2018). 10.1007/978-1-4939-7481-8_7 29224142

[26] Gu, H. et al. Preparation of reduced representation bisulfite sequencing libraries for genome-scale DNA methylation profiling. *Nat. Protoc.* **6**, 468–481 (2011). 21412275 10.1038/nprot.2010.190

[27] Schillebeeckx, M. et al. Laser capture microdissection–reduced representation bisulfite sequencing (LCM-RRBS) maps changes in DNA methylation associated with gonadectomy-induced adrenocortical neoplasia in the mouse. *Nucleic Acids Res.* **41**, e116–e116 (2013). 23589626 10.1093/nar/gkt230 PMC3675465

[28] Wen, L. et al. Genome-scale detection of hypermethylated CpG islands in circulating cell-free DNA of hepatocellular carcinoma patients. *Cell Res.* **25**, 1250–1264 (2015). 26516143 10.1038/cr.2015.126 PMC4650428

[29] Gu, H. et al. Smart-RRBS for single-cell methylome and transcriptome analysis. *Nat. Protoc.* **16**, 4004–4030 (2021). 34244697 10.1038/s41596-021-00571-9 PMC8672372

[30] Bibikova, M. et al. High density DNA methylation array with single CpG site resolution. *Genomics* **98**, 288–295 (2011). 21839163 10.1016/j.ygeno.2011.07.007

[31] Bibikova, M. et al. High-throughput DNA methylation profiling using universal bead arrays. *Genome Res.* **16**, 383–393 (2006). 16449502 10.1101/gr.4410706 PMC1415217

[32] Peters, T. J. et al. Characterisation and reproducibility of the HumanMethylationEPIC v2.0 BeadChip for DNA methylation profiling. *BMC Genom.* **25**, 251 (2024). 10.1186/s12864-024-10027-5 PMC10916044 38448820

[33] Vaisvila, R. et al. Enzymatic methyl sequencing detects DNA methylation at single-base resolution from picograms of DNA. *Genome Res.* **31**, 1280–1289 (2021). 34140313 10.1101/gr.266551.120 PMC8256858

[34] Kresse, S. H. et al. Comparison of enzymatic and bisulfite conversion of circulating cell-free tumor DNA for DNA methylation analyses. *Clin. Epigenetics* **17**, 93 (2025). 40468374 10.1186/s13148-025-01901-4 PMC12135323

[35] Morselli, M. et al. Targeted bisulfite sequencing for biomarker discovery. *Methods* **187**, 13–27 (2021). 32755621 10.1016/j.ymeth.2020.07.006 PMC7855209

[36] Brinkman, A. B. et al. Whole-genome DNA methylation profiling using MethylCap-seq. *Methods* **52**, 232–236 (2010). 20542119 10.1016/j.ymeth.2010.06.012

[37] Aberg, K. A. et al. A MBD-seq protocol for large-scale methylome-wide studies with (very) low amounts of DNA. *Epigenetics* **12**, 743–750 (2017). 28703682 10.1080/15592294.2017.1335849 PMC5739096

[38] Aberg, K. A., Chan, R. F., Xie, L., Shabalin, A. A. & van den Oord, E. Methyl-CpG-binding domain sequencing: MBD-seq. *Methods Mol. Biol.* **1708**, 171–189 (2018). 29224145 10.1007/978-1-4939-7481-8_10

[39] Buchmuller, B. C., Kosel, B. & Summerer, D. Complete profiling of methyl-CpG-binding domains for combinations of cytosine modifications at CpG dinucleotides reveals differential read-out in normal and Rett-associated states. *Sci. Rep.* **10**, 4053 (2020). 32132616 10.1038/s41598-020-61030-1 PMC7055227

[40] Zuccato, J. A. et al. Cerebrospinal fluid methylome-based liquid biopsies for accurate malignant brain neoplasm classification. *Neuro Oncol.* **25**, 1452–1460 (2023). 36455236 10.1093/neuonc/noac264 PMC10398815

[41] Nassiri, F. et al. Detection and discrimination of intracranial tumors using plasma cell-free DNA methylomes. *Nat. Med.* **26**, 1044–1047 (2020). 32572265 10.1038/s41591-020-0932-2 PMC8500275

[42] Moss, J. et al. Comprehensive human cell-type methylation atlas reveals origins of circulating cell-free DNA in health and disease. *Nat. Commun.* **9**, 5068 (2018). 30498206 10.1038/s41467-018-07466-6 PMC6265251

[43] Baca, S. C. et al. Liquid biopsy epigenomic profiling for cancer subtyping. *Nat. Med.* **29**, 2737–2741 (2023). 37865722 10.1038/s41591-023-02605-z PMC10695830

[44] Zeng, Y. et al. A pan-cancer compendium of 1,294 plasma cell-free DNA methylomes and fragmentomes enabling multicancer detection. *Nat. Cancer* **7**, 384–398 (2026). 41714824 10.1038/s43018-026-01116-3 PMC12948677

[45] Stutheit-Zhao, E. Y. et al. Early changes in tumor-naive cell-free methylomes and fragmentomes predict outcomes in pembrolizumab-treated solid tumors. *Cancer Discov.* **14**, 1048–1063 (2024). 38393391 10.1158/2159-8290.CD-23-1060 PMC11145176

[46] Berchuck, J. E. et al. Detecting neuroendocrine prostate cancer through tissue-informed cell-free DNA methylation analysis. *Clin. Cancer Res.* **28**, 928–938 (2022). 34907080 10.1158/1078-0432.CCR-21-3762 PMC8898270

[47] Lu, H. et al. Detection of ovarian cancer using plasma cell-free DNA methylomes. *Clin. Epigenetics* **14**, 74 (2022). 35681212 10.1186/s13148-022-01285-9 PMC9185905

[48] Terp, S. K. et al. Genome-wide cfDNA methylation profiling reveals robust hypermethylation signatures in ovarian cancer. *Cancers* **17**, 2026 (2025). 10.3390/cancers17122026 PMC12190857 40563675

[49] Grisolia, P. et al. Differential methylation of circulating free DNA assessed through cfMeDiP as a new tool for breast cancer diagnosis and detection of BRCA1/2 mutation. *J. Transl. Med.* **22**, 938 (2024). 39407254 10.1186/s12967-024-05734-2 PMC11476115

[50] Wang, J. et al. Altered cfDNA fragmentation profile in hypomethylated regions as diagnostic markers in breast cancer. *Epigenetics Chromatin* **16**, 33 (2023). 37740218 10.1186/s13072-023-00508-4 PMC10517480

[51] Qi, J. et al. Prediction model for malignant pulmonary nodules based on cfMeDIP-seq and machine learning. *Cancer Sci.* **112**, 3918–3923 (2021). 34251068 10.1111/cas.15052 PMC8409309

[52] Ul Haq, S. et al. Cell-free DNA methylation-defined prognostic subgroups in small-cell lung cancer identified by leukocyte methylation subtraction. *iScience* **25**, 105487 (2022). 36425756 10.1016/j.isci.2022.105487 PMC9678731

[53] Qi, J. et al. Plasma cell-free DNA methylome-based liquid biopsy for accurate gastric cancer detection. *Cancer Sci.* **115**, 3426–3438 (2024). 39038922 10.1111/cas.16284 PMC11447983

[54] Zhang, X. et al. Genome-wide analysis of cell-Free DNA methylation profiling with MeDIP-seq identified potential biomarkers for colorectal cancer. *World J. Surg. Oncol.* **20**, 21 (2022). 35065650 10.1186/s12957-022-02487-4 PMC8783473

[55] Luskin, M. R., Murakami, M. A., Manalis, S. R. & Weinstock, D. M. Targeting minimal residual disease: a path to cure? *Nat. Rev. Cancer* **18**, 255–263 (2018). 29376520 10.1038/nrc.2017.125 PMC6398166

[56] Adler, S. et al. Minimum lesion detectability as a measure of PET system performance. *EJNMMI Phys.* **4**, 13 (2017). 28260215 10.1186/s40658-017-0179-2 PMC5337231

[57] Ravera, F. et al. Comprehensive analysis of plasma methylome reveals distinct patterns of methylation changes between responders and non-responders to neoadjuvant chemotherapy in breast cancer. *J. Liq. Biopsy* **6**, 100159 (2024). 10.1016/j.jlb.2024.100159 PMC11863947 40027300

[58] Liu, G. et al. Clinical validation of a tissue-agnostic genome-wide methylome enrichment molecular residual disease assay for head and neck malignancies☆. *Ann. Oncol.* **36**, 108–117 (2025). 39389887 10.1016/j.annonc.2024.08.2348

[59] Peter, M. R. et al. Dynamics of the cell-free DNA methylome of metastatic prostate cancer during androgen-targeting treatment. *Epigenomics* **12**, 1317–1332 (2020). 32867540 10.2217/epi-2020-0173

[60] Burgener, J. M. et al. Tumor-naïve multimodal profiling of circulating tumor DNA in head and neck squamous cell carcinoma. *Clin. Cancer Res.* **27**, 4230–4244 (2021). 34158359 10.1158/1078-0432.CCR-21-0110 PMC9401560

[61] Stutheit-Zhao, E. Y. et al. Clinical validation of a tissue-agnostic genome-wide methylome enrichment assay to monitor response to pembrolizumab. *npj Precis. Oncol.* **10**, 129 (2026). 10.1038/s41698-026-01327-y PMC13013639 41688797

[62] Zuccato, J. A. et al. Prediction of brain metastasis development with DNA methylation signatures. *Nat. Med.* **31**, 116–125 (2025). 39379704 10.1038/s41591-024-03286-y PMC11750707

[63] Maass, K. K. et al. From sampling to sequencing: a liquid biopsy pre-analytic workflow to maximize multi-layer genomic information from a single tube. *Cancers* **13**, 3002 (2021). 10.3390/cancers13123002 PMC8232701 34203921

[64] El Messaoudi, S., Rolet, F., Mouliere, F. & Thierry, A. R. Circulating cell free DNA: Preanalytical considerations. *Clin. Chim. Acta* **424**, 222–230 (2013). 23727028 10.1016/j.cca.2013.05.022

[65] Barra, G. B., Santa Rita, T. H., Jácomo, R. H. & Nery, L. F. Impact of tube additives on baseline cell-free DNA, blood nuclease activity, and cell-free DNA degradation in serum and plasma samples: a comparative study. *LabMed* **2**, 4 (2025).

[66] Danesi, R. et al. What do we need to obtain high quality circulating tumor DNA (ctDNA) for routine diagnostic test in oncology?—considerations on pre-analytical aspects by the IFCC workgroup cfDNA. *Clin. Chim. Acta* **520**, 168–171 (2021). 34081934 10.1016/j.cca.2021.05.033

[67] Shao, J., Nguyen, T. & Li, Z. Impact of long-term plasma storage on cell-free DNA epigenetic biomarker studies. *Biomolecules* **15**, 927 (2025). 10.3390/biom15070927 PMC12292636 40723799

[68] Parpart-Li, S. et al. The effect of preservative and temperature on the analysis of circulating tumor DNA. *Clin. Cancer Res.* **23**, 2471–2477 (2017). 27827317 10.1158/1078-0432.CCR-16-1691

[69] Risberg, B. et al. Effects of collection and processing procedures on plasma circulating cell-free DNA from cancer patients. *J. Mol. Diagn.* **20**, 883–892 (2018). 30165204 10.1016/j.jmoldx.2018.07.005 PMC6197164

[70] Diaz, I. M. et al. Pre-analytical evaluation of streck cell-free DNA blood collection tubes for liquid profiling in oncology. *Diagnostics* **13**, 1288 (2023). 10.3390/diagnostics13071288 PMC10093569 37046506

[71] Lee, J.-S. et al. Clinical practice guideline for blood-based circulating tumor DNA assays. *Ann. Lab. Med.* **44**, 195–209 (2024). 38221747 10.3343/alm.2023.0389 PMC10813828

[72] Dameri, M. et al. Standard Operating Procedures (SOPs) for non-invasive multiple biomarkers detection in an academic setting: a critical review of the literature for the RENOVATE study protocol. *Crit. Rev. Oncol. Hematol.* **185**, 103963 (2023). 36931614 10.1016/j.critrevonc.2023.103963

[73] Sjöström, M. et al. The 5-hydroxymethylcytosine landscape of prostate cancer. *Cancer Res.* **82**, 3888–3902 (2022). 36251389 10.1158/0008-5472.CAN-22-1123 PMC9627125

[74] Zhang, L. & Li, J. Unlocking the secrets: the power of methylation-based cfDNA detection of tissue damage in organ systems. *Clin. Epigenetics* **15**, 168 (2023). 37858233 10.1186/s13148-023-01585-8 PMC10588141

[75] Mondelo-Macía, P., Castro-Santos, P., Castillo-García, A., Muinelo-Romay, L. & Diaz-Peña, R. Circulating free DNA and its emerging role in autoimmune diseases. *J. Pers. Med.* **11**, 151 (2021). 10.3390/jpm11020151 PMC7924199 33672659

[76] Li, S.-J. et al. Cell-free DNA methylation patterns in aging and their association with inflamm-aging. *Epigenomics* **16**, 715–731 (2024). 38869474 10.1080/17501911.2024.2340958 PMC11318736

[77] Sadeh, R. et al. ChIP-seq of plasma cell-free nucleosomes identifies gene expression programs of the cells of origin. *Nat. Biotechnol.* **39**, 586–598 (2021). 33432199 10.1038/s41587-020-00775-6 PMC7610786

[78] Wilson, S. L. et al. Sensitive and reproducible cell-free methylome quantification with synthetic spike-in controls. *Cell Rep. Methods* **2**, 100294 (2022). 36160046 10.1016/j.crmeth.2022.100294 PMC9499995

[79] Francini, E., Nuzzo, P. V. & Fanelli, G. N. Cell-free DNA: unveiling the future of cancer diagnostics and monitoring. *Cancers* **16**, 662 (2024). 10.3390/cancers16030662 PMC10854618 38339412

[80] Francini, E. et al. Circulating cell-free DNA in renal cell carcinoma: the new era of precision medicine. *Cancers* **14**, 4359 (2022). 10.3390/cancers14184359 PMC9497114 36139519

[81] Malapelle, U. et al. Liquid biopsy from research to clinical practice: focus on non-small cell lung cancer. *Expert Rev. Mol. Diagn.* **21**, 1165–1178 (2021). 34570988 10.1080/14737159.2021.1985468

[82] cfDNA Assay Prospective Observational Validation for Early Cancer Detection and Minimal Residual Disease (CAMPERR) (NCT05366881). https://clinicaltrials.gov/study/NCT05366881.

[83] Liu, G. et al. Abstract 2427: the development of a tissue-agnostic genome-wide methylome enrichment MRD assay for applications across the cancer care continuum for head and neck malignancies. *Cancer Res.*10.1158/1538-7445.AM2024-2427https://aacrjournals.org/cancerres/article/84/6_Supplement/2427/736024/Abstract-2427-The-development-of-a-tissue-agnostic (2024).

